# Structural Characterization of BIAN Type Molecules Used in Viscosity Reduction of Alkyl Magnesium Solutions

**DOI:** 10.3390/molecules28020489

**Published:** 2023-01-04

**Authors:** Julia Felicitas Schwarz, Lukas Gadermayr, Samuel Redl, Thorsten Holtrichter-Rößmann, Christian Paulik, Clemens Schwarzinger

**Affiliations:** 1 Institute for Chemical Technology of Organic Materials, Johannes Kepler University (JKU), Altenberger Strasse 69, 4040 Linz, Austria; 2 Institute of Inorganic Chemistry, Johannes Kepler University (JKU), Altenberger Strasse 69, 4040 Linz, Austria; 3 Lanxess Organometallics GmbH, Ernst-Schering-Strasse 14, 59192 Bergkamen, Germany

**Keywords:** magnesium alkyls, viscosity reduction, inert SEC-MS, *N*^1^,*N*^2^-diphenylacenaphthylene-1,2-diimines, structure elucidation, HPLC-MS, FTIR, NMR

## Abstract

*N*^1^,*N*^2^-diphenylacenaphthylene-1,2-diimines (BIANs) have been used to reduce the undesired high viscosity of alkyl magnesium solutions, which are known to form polymeric structures. In order to understand the mechanisms, analyses of the BIAN alkyl magnesium solutions have been carried out under inert conditions with SEC-MS, NMR, and FTIR and were compared to the structures obtained from HPLC-MS, FTIR, and NMR after aqueous workup. While viscosity reduction was shown for all BIAN derivatives used, only the bis (diisopropyl)-substituted BIAN could be clearly assigned to a single reaction product, which also could be reused without loss of efficiency or decomposition. All other derivatives have been shown to behave differently, even under inert conditions, and decompose upon contact with acidic solvents. While the chemical reactions observed after the workup of the used BIANs are dominated by (multiple) alkylation, mainly on the C = N double bond, the observation of viscosity reduction cannot be assigned to this reaction alone, but to the interaction of the nitrogen atoms of BIANs with the Mg of the alkyl magnesium polymers, as could be shown by FTIR and NMR measurements under inert conditions.

## 1. Introduction

Organomagnesium compounds find important use in the metalation of aromatic compounds, the alkylation of metal oxides or halides, and ketone reduction [1,2,3,4]. Furthermore, they are important in the application of polymerizations, such as the production of polyethylene, polypropylene, polyethylene glycol [5], production of telomers [6] and conjugated dienes, such 1,3-butadiene and isoprene.

Dialkyl magnesium compounds represent one of the most important organomagnesium substance groups [2]. Nevertheless, dialkyl magnesium compounds are not as common as Grignard reagents (RMgX, X = Cl^−^, Br^−^, or I^−^), although they perform similar reactions [7]. In the past, a reason for this was the lack of an industrially relevant synthesis route to produce dialkyl magnesium compounds in large quantities [7,8].

In the meantime, certain dialkyl magnesium compounds can be produced on a large scale by reacting magnesium powder with the alkyl halides in hydrocarbon solvents [2,7]. Especially, branched-chain alkyl groups, cyclic alkyl groups, or the linear residues of five or more carbons are available, but there are also particular combinations of shorter-length alkyl chains, such as butyl ethyl magnesium, which are all soluble in hydrocarbons [2]. Alkyls, such as *n*-butyl *sec*-butyl magnesium and di-*sec* butyl magnesium, are also readily soluble in hydrocarbon solvents, but they have to be produced via lithium alkyls and are, therefore, much more expensive [7,9].

With the discovery of magnesium chloride supported Ziegler–Natta catalysts, magnesium alkyls gained additional importance. Especially the specific morphology and structure of the MgCl_2_ is a key parameter for highly active Ziegler–Natta catalysts [10,11,12,13,14,15]. Magnesium alkyls are one of the most important feedstocks for the large-scale production of magnesium chloride, as they combine the advantages of being available in inert solvents, such as hydrocarbons, and are, thus, free of water and ether. Major benefit is that the resulting solutions can be handled more easily than moisture-sensitive solids [16,17,18,19].

The major problem of dialkyl magnesium in hydrocarbon solvents is their high viscosity, which poses a significant problem in processibility [2,4]. The most relevant magnesium alkyls in industry are butyl octyl magnesium (BOMAG) and butyl ethyl magnesium (BEM), being sold in 10–20% solutions [1,20]. This concentration is the optimum between accessible magnesium, industrial processability, viscosity, and economic factors [20].

**Figure 1:**
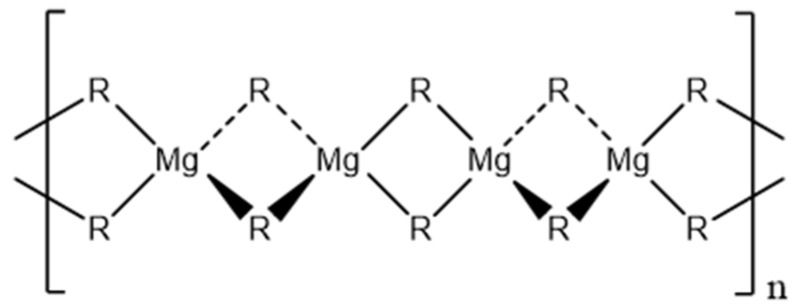
Polymeric chain structure of dialkyl magnesium compounds [20,22,24].

An explanation for the high viscosity is the formation of a polymeric structure via two-center three electron bonding, whereby the magnesium atom is tetrahedrally surrounded by four alkyl groups (Figure 1) [20,21,22], which is based on the X-ray crystal structure of dimethyl magnesium [23].

As higher concentrations of alkyl magnesium solutions are desirable for economic and technical reasons, the reduction of their viscosity is a key target of industrial research. However, only a few examples for viscosity modifiers are described in the literature, including organometallic compounds of gallium, indium or lithium [2], benzene derivates [25], and organoaluminum compounds [26]. From these groups, the latter ones are the only ones used on a large scale [20,26,27]. Too high aluminum concentrations, however, cannot be used, as they lead to catalyst poisoning [28]. New research identified heterocumulenes as highly effective viscosity modifiers, particularly carbodiimides, such as dicyclohexyl carbodiimide or trimethylsilyl carbodiimide [29,30]. Structural features of the reaction of heterocumulenes with dialykl magnesium compounds in donating solvents, such as ethers, were studied by Chlupatý et al. and Anga et al.; however, equimolar ratios were used in their studies [31,32]. The structures and influence of the heterocumulenes and other viscosity modifiers with magnesium alkyls remained unexplored. Only little data on the structural elucidation of highly reactive water and air sensitive organometallic compounds are available, e.g., by using mass spectrometry for methyl aluminoxanes [33,34].

It is the major goal of this work to investigate the interaction of *N*^1^,*N*^2^-diphenylacenaphthylene-1,2-diimines (BIANs) with alkyl magnesium compounds in toluene to reduce the solutions’ viscosities. By using various analytical techniques, such as FTIR, NMR, and mass spectrometry, under inert conditions, we have tried to identify a stable compound that does not interfere with future applications of the alkyl magnesium solutions. Furthermore, the reaction products obtained after aqueous workup were isolated and identified, as well as tested for changes in their viscosity-reducing properties.

## 2. Results and Discussion

### 2.1. Effect of BIANs on Viscosity of BOMAG

**Scheme 1:**
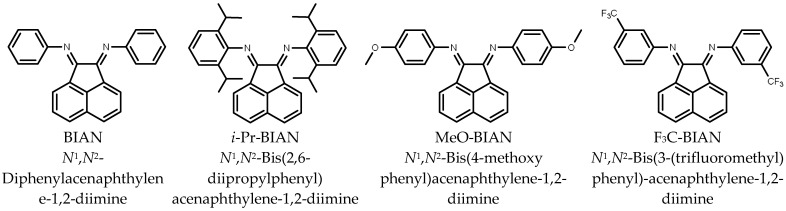
Structures of the BIAN derivatives used in this study.

Four BIAN compounds were selected for the viscosity modification of alkyl magnesium solutions (Scheme 1). An unsubstituted BIAN (*N*^1^,*N*^2^-diphenylacenaphthylene-1,2-diimine); (abbreviation: BIAN), BIAN with isopropyl groups in ortho positions (*N*^1^,*N*^2^-bis(2,6-diisopropylphenyl)acenaphthylene-1,2-diimine; abbreviation: *i*-Pr BIAN), with methoxy groups in para position (*N*^1^,*N*^2^-bis(4-methoxyphenyl)acenaphthylene-1,2-diimine; abbreviation: MeO-BIAN), and with trifluoromethyl groups in meta position (*N*^1^,*N*^2^-bis(3-(trifluoromethyl)phenyl)acenaphthylene-1,2-diimine; abbreviation: F_3_C-BIAN). The viscosity reduction effect was measured for BOMAG in solutions in heptane and in toluene.

The reactions between the BIANs and BOMAG were all exothermic, and the colorless BOMAG solution changed its color from a red to a purple solution (Table 1) in all cases. The additives showed a drastic viscosity reduction of about 70% in both solvents (Table 1). The viscosities in toluene are always higher, since the initial viscosity of BOMAG is already higher in toluene. The BIAN substitution effect is of negligible importance on the viscosity-reducing effect, except for F_3_C-BIAN. In the latter case, only 50% BIAN are present, and 50% of the singly substituted monoimine comprise only one C = N double bond (see NMR measurements of all pure BIANs in Figures S1–S7). This is also in good agreement with the lower viscosity reduction of 50%.

### 2.2. SEC and SEC-MS Analysis under Inert Conditions

As alkyl magnesium compounds are extremely sensitive to water and all protic solvents, a chromatographic separation of their solutions is not possible with standard equipment. In our effort to perform size exclusion chromatography (SEC) analyses, we have transferred the SEC system into a mobile fume hood that was flushed with nitrogen to create an inert atmosphere. The solvent, toluene, was dried over sodium directly before use to ensure absolute dry conditions. Seal washing and syringe washing solutions have also been exchanged to absolute toluene. The samples were prepared in a glove box and measured as quickly as possible.

**Figure 2:**
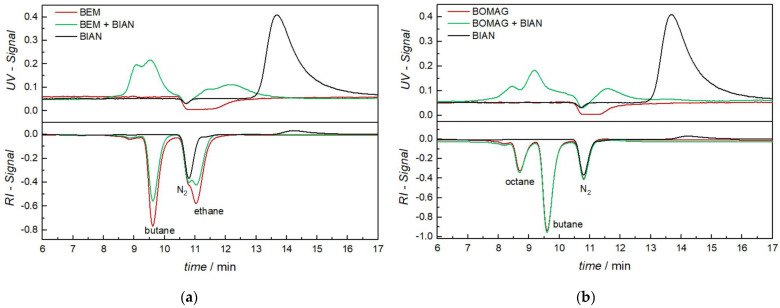
Size exclusion chromatograms using UV (**top**) and RI (**bottom**) detection: (**a**) analysis of BIAN (black), BEM (red), and the mixture of BEM with BIAN (green) and (**b**) BIAN (black), BOMAG (red), and the mixture of BOMAG with BIAN (green).

As a first example, we will discuss unmodified BIAN and its interaction with BEM, as well as BOMAG. Figure 2 shows the chromatograms of pure BIAN obtained with a UV detector (upper black trace) and with an RI detector (lower black trace). In UV, only the intact BIAN molecule is visible with a retention time of 13.7 min (peak apex). In the RI detector, a negative peak is visible at 10.8 min, which is due to the dissolved nitrogen in the sample solution. The BIAN signal is found with an apex of 14 min, as the RI detector is located after the UV detector. The expected separation range of the chosen SEC column is typically between 6 and 8 min, as has been determined with polystyrene standards between 370 and 6600 g mol^−1^; thus, the high retention time of BIAN indicates a strong adsorption on the column material using toluene as solvent and, thus, a separation mode that is not only dependent on size.

Analysis of pure BEM (Figure 2a, red trace) does not show any response in the UV detector, except for a broad negative signal between 11 and 12.5 min, which cannot be explained, even with mass spectrometric detection. The RI detector shows three negative signals, from which one is centered at 10.8 min and, thus, assigned to nitrogen, with the others being butane (9.6 min) and ethane (11.0 min). Those gases are formed in the reaction of BEM with a proton source, indicating either incomplete inertness of the SEC system or a catalytic reaction with some part of the injection system or column compartment. As later investigations have shown, Mg(OH)_2_ could be identified with FTIR spectroscopy on the inlet filter of the SEC column, indicating that the decomposition already takes place before the sample enters the analytical column. Despite all efforts to exclude moisture any further, those signals remained and showed that part of the sample is degraded during the measurements. Figure 2b shows the analysis of pure BOMAG (red). While the UV is identical to BEM, the RI trace now shows three well-separated peaks, from which two, nitrogen (10.8 min) and butane (9.6 min), are already known. The third one at 8.7 min can be assigned to octane.

When BIAN is mixed with BEM or BOMAG the chromatograms obtained from the RI detector remain unchanged, the only small difference is that the shallow BIAN signal at 14 min vanishes. Looking at the UV traces, a big difference can be observed. Again, the BIAN signal (13.7 min) is completely gone, but new signals in the range of 7.6–10 min and 11–12.5 min can be found. Combing these two observations gives a clear indication that, despite a partial decomposition of the alkyl magnesium compounds, a chemical interaction between them and the BIAN takes place. Given the fact that the newly formed compounds all elute before the original BIAN, it can be deduced that these structures are either larger (size exclusion chromatography mechanism) or less polar (adsorption chromatography mechanism).

**Figure 3:**
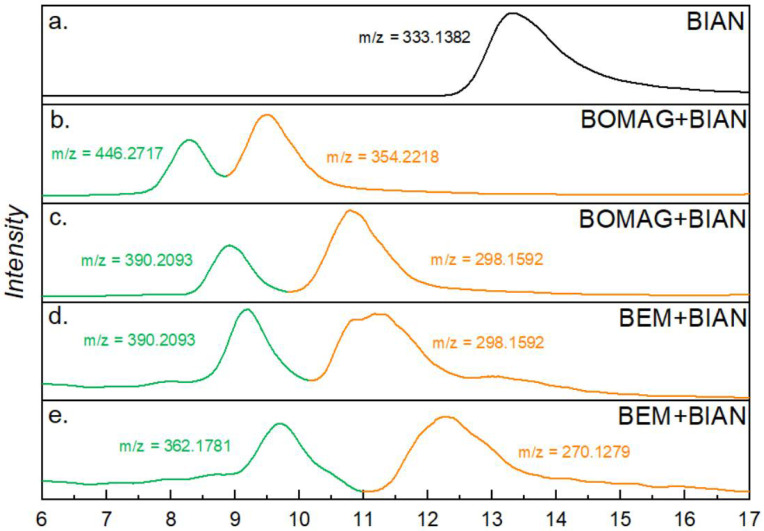
SEC-MS results of BIAN (**a**), BIAN with BEM (**b**,**c**), and BIAN with BOMAG (**d**,**e**); the traces show chromatograms of extracted *m*/*z* values, and the corresponding structures can be found in Table 2.

To identify the newly formed products we have coupled the SEC system to a high resolution Orbitrap mass spectrometer. This was accomplished by splitting the eluent before the RI detector (to prevent over pressure in the optical cell) and inserting an about 70 cm long PEEK tubing to transfer the samples into the APCI source of the mass spectrometer. In a first run, pure BIAN was injected to verify whether the ionization process with an APCI source and toluene as eluent works efficiently. A single broad peak at 13.4 min retention time was found, and the mass signal was obtained with *m*/*z* = 333.1382, corresponding to a singly protonated BIAN with a theoretical mass of *m*/*z* = 333.1386 and, thus, only 1.2 ppm deviation (Figure 3a).

Analysis of the BIAN + BEM sample resulted in two major signals that overlapped with the first two UV signals and could be assigned to butyl- and ethyl-substituted BIAN; however, the *m*/*z* values indicate the species to be radical cations. This assumption is also supported by MS results using negative ionization, which shows [M-H]^−^ ions. The alkyl group is most likely attached to the carbon of a C = N bond (Table 2). Furthermore, at a retention time between 11 and 12.5 min, two signals were found, this time with the proposed structure of an alkyl group substituting the aniline (Table 2), and in this case, MH^+^ ions were detected (Figure 3d,e). When BEM is substituted by BOMAG, both butyl substituted species could be found again, as well as those that are substituted with an octyl group (Figure 3b,c, Table 2).

When doing the same type of analysis with the substituted BIANs shown in Scheme 1, a similar behavior was found for MeO-BIAN and F_3_C-BIAN. Again, upon mixing them with BEM, one species was found where the butyl and ethyl groups were added to the double bond of the intact BIAN and a second species, where the alkyl groups replaced one of the anilines. Reactions with BOMAG showed the same two structures, but this time with butyl and octyl residues, respectively. Chromatograms and *m*/*z* values can be found in the Supplementary Materials (Figures S8–S13 and Tables S1 and S2). i-Pr-BIAN is the only species that shows a somewhat different behavior. Although, again, a structure bearing an attached alkyl group could tentatively be identified, it must be noted that, in this case, that it was not radical cations that were observed, but regular MH^+^ ions (Figures S14 and S15, Table S3). The second species, with one aniline removed could not be found with i-Pr-BIAN.

### 2.3. NMR Analysis of Inert Dibutyl Magnesium BIAN Mixtures

To find out if the structures identified with the SEC-MS in toluene are reactive species or can be isolated, all BIANs were reacted with dibutyl magnesium. The latter was chosen as it has only one alkyl group, thus the number of formed products should be reduced, enabling easier identification. BIANs and dibutyl magnesium solution were thoroughly mixed and then carefully quenched with water. During the exothermic reaction, the color remained violet/dark red until all of the alkyl magnesium was destroyed. At this point, the color changed to orange/yellow for all BIANs. This was interpreted in a way that the interaction between the alkyl magnesium and the BIANs was very strong, and the intermediates/complexes formed were stable until fully quenched. The organic phase was then isolated, dried under reduced pressure, and analyzed with HPLC-MS, FTIR, and NMR.

### 2.4. HPLC-MS Measurments of Quenched Dibutyl Magnesium BIAN Mixtures

**Figure 4:**
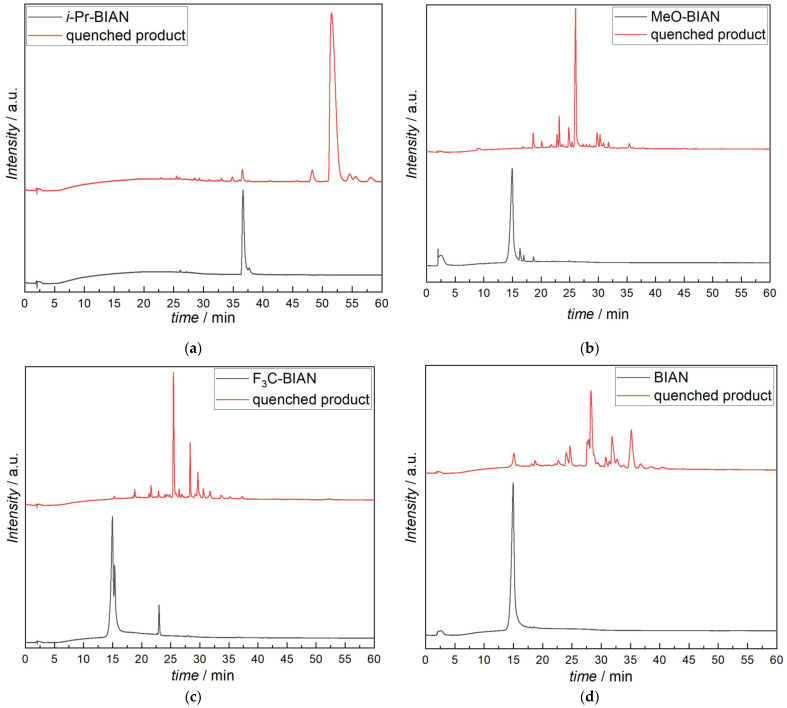
HPLC-MS analysis of the BIANs before and after reaction with dibutyl magnesium: (**a**) *i*-Pr-BIAN, (**b**) MeO-BIAN, (**c**) F_3_C-BIAN and (**d**) BIAN; the corresponding structures can be found in Table 3 and Scheme S1.

In a first step, the pure BIANs were analyzed by HPLC-MS, and BIAN, MeO-BIAN and F_3_C-BIAN all showed the same behavior, i.e., that, although being dissolved in dry acetonitrile, they decomposed as soon as they came into contact with the acidic buffer system (acetonitrile/water/formic acid). The products found were acenaphthoquinone and the corresponding aniline or substituted anilines (Figure 4b–d). i-Pr-BIAN was the only derivative that could be analyzed without decomposition, and it showed a single peak (36.5 min) with an *m*/*z* value corresponding to the intact molecule (MH^+^ ion) (Figure 4a).

The quenched BIANs, however, showed very different results. The simplest example is, again, i-Pr-BIAN, showing only one major reaction product, which is the singly butyl-substituted i-Pr-BIAN (51.5 min) (Figure 4a). Additionally, small amounts of remaining i-Pr-BIAN (36.6 min) were found, as well as one compound that had a butyl group attached, but must have an additional double bond, according to the exact mass of 557.3887 Da (48.3 min). Within this structure, the double bond is situated exactly cannot be identified from the mass spectrum alone (Table 3).

The reaction products of MeO-BIAN are not so uniform (Figure 4b). Again, the most intense peak can be assigned to a singly butylated BIAN structure, but in this case, a small amount of the degradation product of the pure MeO-BIAN (methoxy aniline and acenaphtoquinone) was also found, indicating that a certain amount of the MeO-BIAN had not irreversibly reacted with the alkyl magnesium compound. The structures which had further been identified were N-butyl aniline, two isomers of a doubly butylated BIAN bearing an additional double bond, and one doubly butylated species with two additional double bonds (Table 3).

F_3_C-BIAN (Figure 4c) gives even more reaction products. Again, the singly butylated BIAN is found, but only in small amounts. Dominating species are ones where one aniline is substituted by a butyl group and the other aniline is cleaved off leaving a carbonyl group behind, as known from the pure BIANs, as well as another one with two butyl groups and one aniline removed. Of particular interest is the detection of *N*-butyl trifluoromethylaniline, which shows that, although only in small amounts, alkylation of the nitrogen occurs. If the alkylation takes place on the intact BIAN or already on the cleaved off aniline cannot be determined from the data available. A list of all compounds found in the HPLC-MS analysis can be seen in Table 3 and Scheme S1.

Unsubstituted BIAN (Figure 4d) yields the most complex mixture upon reaction with dibutyl magnesium. While, again, the most intense peak can be assigned to the butyl substituted BIAN, a good amount of aniline and acenaphtoquinone are also found, indicating incomplete turnover. Other compounds present in larger quantities have been identified as the butyl substituted monoketone (as already found in F_3_C-BIAN), dibutyl-BIAN, and several other alkylated BIAN derivatives (tentative structures can be found in Table 3 and the Supplementary Materials Scheme S1). In contrast to all other BIANs, it was possible to identify two isomers of a BIAN with two butyl groups attached, from which one must bear a butyl group on a nitrogen atom (entry 37 in Table 3). This assignment is performed on basis of the MS/MS fragmentation analysis, which clearly shows that a butyl aniline is cleaved off the molecular ion, while, in the case of the other isomer (entry 36 in Table 3), aniline is cleaved off.

### 2.5. NMR-Measurments of Inert and Quenched Dibutyl Magnesium BIAN Mixtures

NMR of the inert samples of dibutyl magnesium and its mixture with BIANs have been recorded to further investigate the active species. i-Pr-BIAN was chosen as reference, as it showed only one reactive intermediate in the SEC-MS experiments (Figures S16 and S17). While it is possible to easily identify the aromatic peaks of the BIAN [35,36,37,38], the alkyl region is extremely crowded. A couple of sharp alkyl signals at 0.6, 1, 1.4, and 1.5 ppm are visible, too, which can be assigned to the newly formed butyl BIAN derivative found in the SEC-MS experiments. A clear assignment, however, is not possible, as both the ^1^H, but especially the ^13^C, spectra are very crowded and the alkyl groups of the reaction product and the alkyl magnesium overlap. A possible reason might be the formation of a complex between Mg and the BIAN, as found for Mg and carbodiimides [31], which cannot be detected in SEC-MS, due to dissociation in the ionization process. One signal that can be assigned in the ^13^C spectra is the carbon of the C = N double bond at 160.9 ppm in native i-Pr-BIAN, which shifts to 194.5 ppm in the inert i-Pr-BIAN dibutyl magnesium mixture and after aqueous workup back to 175.2 ppm. This shift indicates a change in the electron density of this bond, especially under inert conditions, which is similar to the shift from 140 of dicyclohexylcarbodiimide to 181.7 ppm for the complex of dicyclohexylcarbodiimide with dibutyl magnesium observed by Chlupaty et al. [31].

**Figure 5:**
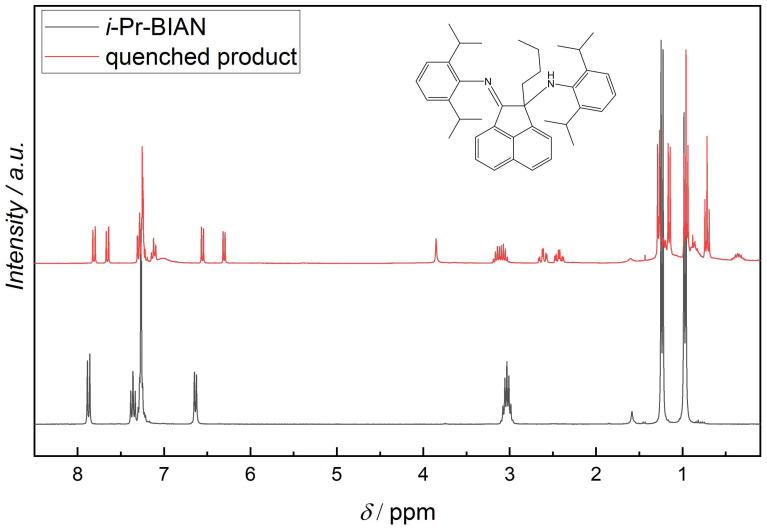
^1^H-NMR of *i*-Pr-BIAN and the extracted product from the reaction of *i*-Pr-BIAN with dibutyl magnesium after water quenching in CDCl_3_.

**Figure 6:**
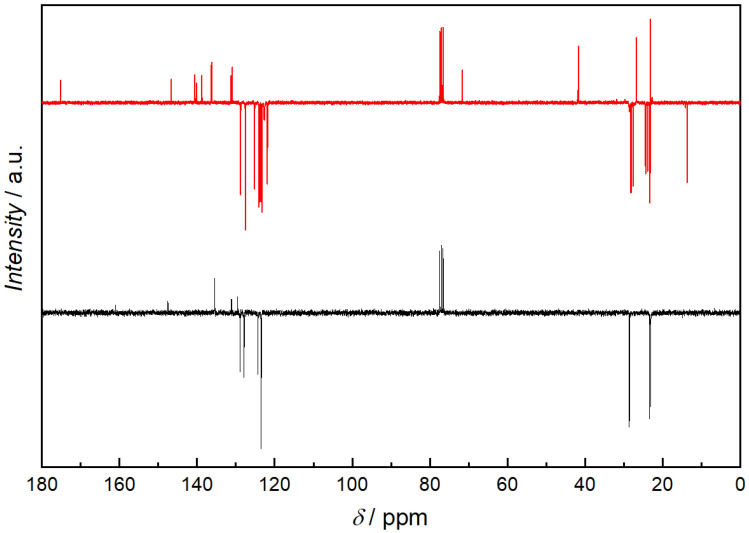
APT ^13^C-NMR spectra of *i*-Pr-BIAN (black) and the extracted product from the reaction of *i*-Pr-BIAN with dibutyl magnesium after water quenching (red) in CDCl_3_. Spectra are displayed upside down (quaternary and CH_2_ signals are displayed below the baseline and CH_3_ and CH signals can be found above the baseline).

**Figure 7:**
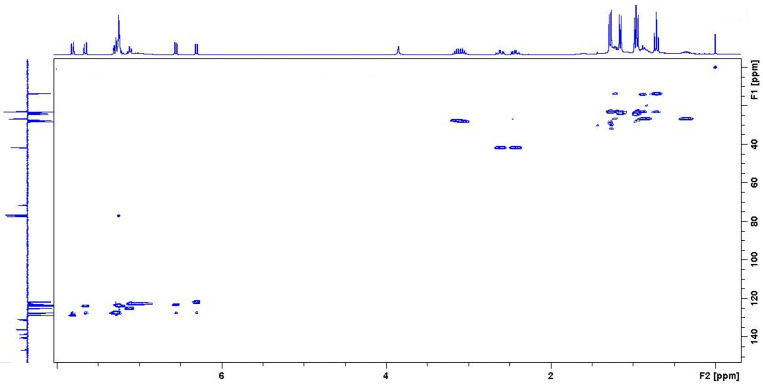
HSQC of the quenched *i*-Pr-BIAN product in CDCl_3_.

NMR measurements of the quenched samples were carried out, in order to understand the structure of the resulting compounds. Since the quenched i-Pr-BIAN product is the purest, it will be discussed here in more detail. In addition to a ^1^H-NMR (Figure 5) and an APT (Figure 6), a HSQC-NMR was carried out (Figure 7).

Using the HPLC-MS results as starting point, an alkylated i-Pr-BIAN is proposed as a structure. The major question to be solved via NMR is the position of the alkyl group. Comparison of the ^1^H-NMRs of i-Pr-BIAN and the quenched product (Figure 5) shows a new signal at 3.85 ppm assigned to an NH-group and a splitting of aromatic, as well as alkyl signals. The splitting can be explained by the fact that the addition of the alkyl group to the C = N bond the aniline moieties are no longer symmetrical. Additionally, a triplet at 0.7 ppm, indicative of the CH_3_-group of an alkyl chain, can be seen, which is assigned to the butyl group. Another good indicator for the structure proposed in Figure 5 can be found in the HSQC spectrum (Figure 7), as the proton at 3.85 ppm is not attached to any carbon and, therefore, must be attached to a nitrogen atom. In the APT spectrum (Figure 6), all signals pointing up are quaternary carbons, except the carbons at 41.8, 26.8, and 23.2 ppm, which are the CH_2_ groups of the butyl group. Again, this can only be explained if the butyl group is attached to the newly formed quaternary carbon at 71.7 ppm. The quaternary C = N carbon in the initial substance is at 160.9 ppm and shifts to 175.2 ppm in the extracted product, as already described above. Again, as seen in the ^1^ H-NMR spectrum, the loss of symmetry leads to more chemically different carbons in the ^13^ C-NMR (Figure 6) and, therefore, a higher number of signals.

The shift of the C = N carbon to higher ppm values can be observed in all other BIANs, too, as well as the formation of a new quaternary carbon atom, confirming the alkylation of the C = N carbon. Further interpretation of the spectra was not performed, as the quenched products of BIAN, MeO-BIAN, and F_3_C-BIAN are all complex mixtures (Figures S18–S21).

### 2.6. FTIR-Analysis of Quenched Dibutyl Magnesium BIAN Mixtures

**Figure 8:**
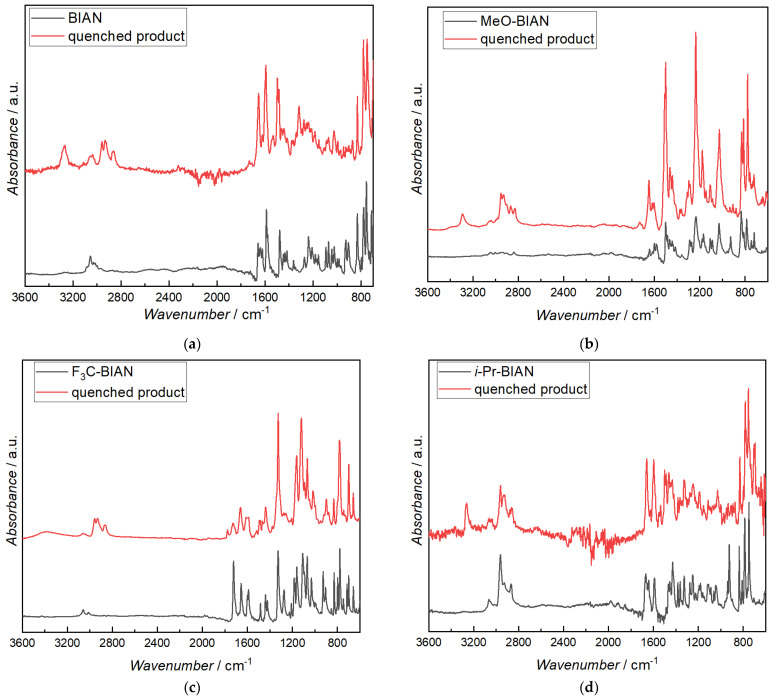
FTIR spectra of pure BIANs (black) and BIANs reacted with dibutyl magnesium (red): (**a**) BIAN, (**b**) MeO-BIAN, (**c**) F_3_C-BIAN, and (**d**) *i*-Pr-BIAN.

FTIR analysis of the isolated compounds was performed mainly to prove that the alkylation of the BIANs took place on the carbon of the C = N double bond. Unreacted BIAN shows aromatic C-H bands at 3000–3100 cm^−1^, and, after quenching, aliphatic C-Hs from the alkyl chain are visible between 2800–3000 cm^−1^, as well as in intense band at 3270 cm^−1^, which can be assigned to the N-H valence vibration of a secondary amine. In the fingerprint range a band at 1650 cm^−1^ becomes more intense, and two at 1495 cm^−1^ and 1318 cm^−1^ evolve (Figure 8a). The latter one can also be assigned to the N-H vibration, confirming the alkylation of the BIAN at the carbon atom of the double bond. The 1640 and 924 cm^−1^ bands, which are assigned to the C = N valence and deformation, are significantly lower in the quenched BIAN, also indicating reaction on one of the two C = N bonds [39,40].

As already known from the discussion of BIAN, the quenched i-Pr-BIAN shows a distinct secondary N-H band at 3265 cm^−1^, and the broad C-N deformation band at 700 cm^−1^ is also visible. Aromatic, as well as aliphatic, C-H bands remain more or less unchanged as the unreacted i-Pr-BIAN already contains 4 isopropyl groups, resulting in the 2800–3000 cm^−1^ bands. When looking closely, however, an increase of the aliphatic CH_2_ band (2932 cm^−1^), relative to the CH_3_ band (2964 cm^−1^), is visible. The C = N deformation band at 926 cm^−1^ is, again, significantly lowered in the quenched i-Pr-BIAN (Figure 8d).

In MeO-BIAN (Figure 8b), the N-H valence band is seen at 3295 cm^−1^, together with an increase in the CH valence bands between 2800 and 3000 cm^−1^. Again, the band at 927 cm^−1^, indicative for a C = N bond, almost disappears, indicating a reaction at this position. It is also noted that there is an increased absorption at around 1750 cm^−1^, due to the formation of a carbonyl bond in the MeO- and F_3_C-BIAN, which is in good agreement with data obtained from the HPLC-MS experiments.

While the pure F_3_C-BIAN only shows signals from aromatic C-Hs (3000–3100 cm^−1^), the quenched compound clearly shows aliphatic C-H bands from the alkyl group (2800–3000 cm^−1^) and an additional broad band centered at 3400 cm^−1^ (Figure 8c). The latter one is a bit too high for a secondary amine, as they have been found in all other BIANs, and it is also much broader. The C = N band at 929 is again lowered dramatically, as already known from the other quenched BIANs. While the CF_3_ bands at 1327 and 782 cm^−1^ remain unchanged, a series of other bands become broader and of lower intensity, e.g., the ones at 1722, 1656, 1600, 1590, and 1275 cm^−1^, as well as the one at 1275 cm^−1^. The ones at 1423, 1186, 1031, and 716 seem to be missing at all. As HPLC analysis has already shown, the quenched F_3_C-BIAN is composed of many individual breakdown products; thus, the interpretation of the IR spectrum is not focused on any further products.

### 2.7. FTIR-Analysis of an i-Pr-BIAN BOMAG Solution

**Figure 9:**
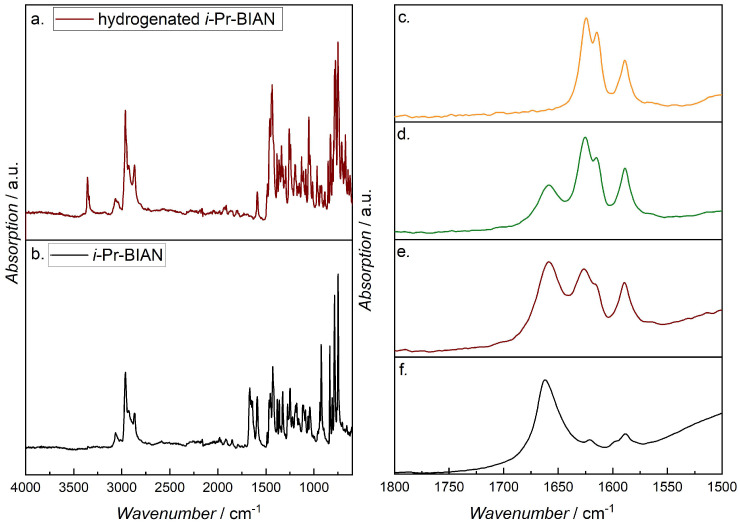
FTIR spectra of *i*-Pr-BIAN (**a**) and the fully hydrogenated *i*-Pr-BIAN (**b**); details of the FTIR spectra of the *i*-Pr-BIAN BOMAG heptane solution under (**c**) argon atmosphere, (**d**,**e**) after removal of the argon stream, and (**f**) after completed reaction.

In order to prove the hypothesis that the metal of the alkyl magnesium polymers interacts with the C = N double bond of the BIANs, experiments under argon atmosphere have been performed. At first, the assignment of the C = N band at 1650 cm^−1^ was verified by comparing the spectrum of i-Pr-BIAN with its fully hydrogenated derivative (Figure 9a,b). The NH band of the hydrogenated i-Pr-BIAN can be seen at 3360 cm^−1^, and no band at 1665 cm^−1^, as in i-Pr-BIAN, was found, confirming the assignment of the latter to the C = N double bond. For the inert measurements of the i-Pr-BIAN BOMAG mixture in heptane, a one-inch diameter polymer cylinder was placed around the diamond crystal of the ATR unit, and the setup was flushed with argon from the top during collection of background and spectra. The i-Pr-BIAN BOMAG heptane solution was transferred to the FTIR spectrometer by means of an argon flushed syringe, a drop of the solution was placed directly on top of the ATR crystal, and the first spectrum was recorded under a stream of argon. In order to minimize the measurement time, the number of scans was reduced to 8. After this first spectrum was recorded, the argon stream was removed, and the mixture started to react with air. While the initially deep red solution changed more and more into an off-white solid, additional FTIR spectra were recorded (Figure 9c–f).

Figure 9c shows the 1500–1800 cm^−1^ region of the spectrum of the i-Pr-BIAN and BOMAG in heptane solution under argon, where a double band at 1624 and 1614 cm^−1^ is visible, but there is none at 1670 cm^−1^, as seen in i-Pr-BIAN, or 1664 cm^−1^, as in the quenched (alkylated) i-Pr-BIAN (compare to Figure 8d). Such shifts to lower wavenumbers have already been reported for C = N double bonds interacting with platinum [41], palladium [42], ands chromium [43], as well as iron, cobalt, and copper [44], are attributed to a decrease in the C = N bond order, due to the coordination of the metal with the imine lone pair, thus proving the interaction of the magnesium in the BOMAG chains with the C = N bind of the BIAN.

When the sample starts to react with moisture after removal of the argon stream, the spectra show the evolution of the 1660 cm^−1^ band, indicating the destruction of the Mg–imine complex. With an increase in time, the ratio of the 1660/1624 (1614) bands becomes larger, until finally, only the 1660 cm^−1^ band remains (Figure 9d–f). Despite this change, the formation of Mg(OH)_2_, indicated by a band at around 3720 cm^−1^, can be seen. Once no further reaction can be observed, the off-white powder was extracted with acetonitrile, and the solution subjected to HPLC-MS analysis, where the reaction product could again be identified as alkylated i-Pr-BIAN, in this case with butyl and octyl residues.

### 2.8. Reusability of Quenched BIANs

The BIANs that have been reacted with dibutyl magnesium and then quenched with water were isolated, as described in the HPLC section, and reused as viscosity modifiers. Again, all the tested products reduced the viscosity of a BOMAG solution in toluene. The effect of the reaction products of MeO-BIAN and F_3_C-BIAN was much smaller, and the viscosity dropped by only about 40%, instead of the original 70% (Table 1). For both of these substances, the HPLC-MS measurement demonstrated that many different compounds were formed from the native BIANs (Figure 4), which presumably exhibit no viscosity-reducing effect, but on the other hand, might pose a problem for the further use of the BOMAG solution. Still, very good results were obtained for the reaction product of i-Pr-BIAN, which is essentially the butylated i-Pr-BIAN (Figure 5).

**Figure 10:**
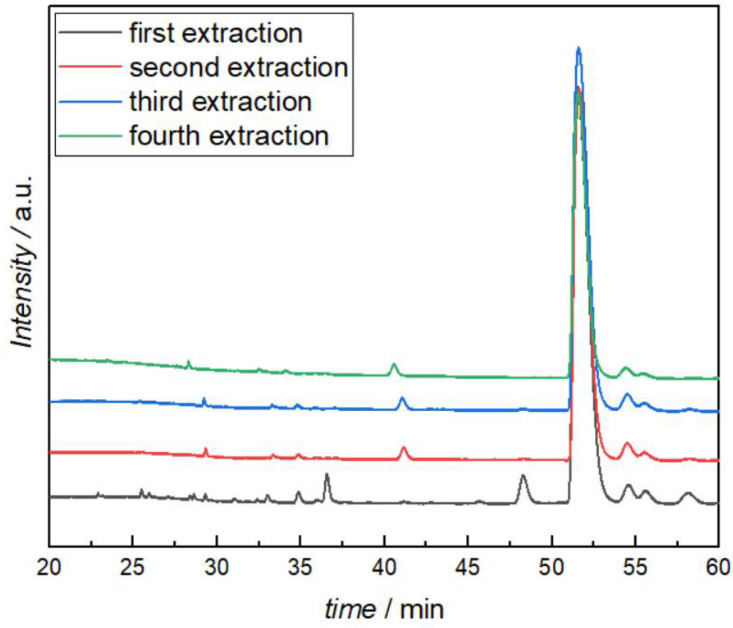
HPLC analysis of the quenched *i*-Pr-BIAN after reaction with dibutyl magnesium (first extraction) and after additional viscosity measurements and extractions.

After measuring the viscosity with the above-mentioned products (Table 4), the reaction mixtures were again quenched with water, extracted, and analyzed with HPLC-MS to find out if the structures have changed. No major changes in any of the BIAN reaction products were observed. The butylated i-Pr-BIAN was even reused as a modifier two more times with subsequent HPLC-MS analysis, which showed no significant changes between each use and extraction cycle (Figure 10). From the last reuse experiment, no viscosity could be measured, as the amount of sample after workup was already too small.

## 3. Experimental

All reactions were performed under inert conditions using Schlenk technique in an argon (5.0, Linde) atmosphere or in a nitrogen-filled M. Braun glove box. The magnesium alkyls butyl octyl magnesium (BOMAG), and butyl ethyl magnesium (BEM) in toluene or heptane were provided by LANXESS Organometallics GmbH and di-*n*-butyl magnesium (1 M in heptane) was obtained from Sigma Aldrich. Toluene (rotisolv^®^, Roth) was further dried over sodium in argon atmosphere. The BIAN additives were synthesized by ourselves, according to the literature [35,36,37,38].

### 3.1. Viscosity Reduction of Alkyl Magnesium Compounds

For the viscosity modification around 9 g of BOMAG solution (19.3% in toluene, or 20.4% in heptane) were used, and approximately 2.5 mol% (relative to the Mg content) additive were added at room temperature. After stirring for 5 min, the temperature was raised to 50 °C for half an hour.

After cooling down to room temperature, the viscosity was measured using a DV2T Brookfield spindle viscosimeter. The measurement was performed under an argon stream at 21 °C and 40 rpm with a SC4-18 spindle. Depending on the viscosity, the rotational speed was adjusted.

### 3.2. Synthesis and Reusability of Reaction Products with Dibutyl Magnesium

Approximately 4–5 mL of dibutyl magnesium were mixed with 10 mol% of the respective BIAN derivative. Then, 5 mL heptane were added, and the solution slowly mixed with water. During the exothermic reaction, the alkyl magnesium is destroyed in a controlled manner, and magnesium hydroxide is formed. The violet-reddish colors of the initial mixture remained until the end, and after all the magnesium alkyl had reacted, the color changed to orange/yellow.

The organic phase was separated from the aqueous phase, toluene was removed under reduced pressure, and the resulting solid was dried in vacuo. The products were then analyzed using FTIR, HPLC-MS, and NMR. Further, the newly formed compounds or mixtures were tested again as viscosity modifiers, as described in Section 3.1.

### 3.3. Analytical Methods

#### 3.3.1. NMR

The collection of the NMR data was achieved using an Avance III (Bruker, Birica, MA, USA) spectrometer (300 MHz). For air and water sensitive compounds, benzene-d6 (deutero, 99%) was used, and for the pure BIANs and extracted products, CDCl_3_ (Roth, 99.8%, with TMS) was chosen.

#### 3.3.2. SEC and SEC-MS

Size exclusion chromatography was performed on a Wyatt SEC system consisting of an Agilent 1260 isocratic pump, Agilent 1260 VWD UV-Vis detector, Agilent 1260 Autosampler, Shodex RI-501 refractive index detector, and a Wyatt Treos II light scattering detector. Samples were dissolved in toluene in a glove box at a concentration of around 1 mg mL^−1^ and 50 µL injected and separated on a Phenogel 50 A column (300 × 4.6 mm, 5 µm; Phenomenex) using 0.35 mL min^−1^ toluene as mobile phase. To exclude moisture, the complete system was placed in a mobile fume hood and flushed with dry nitrogen. Additionally, a beaker filled with water absorbing silica gel was placed in the autosampler compartment. The solvent was dried over sodium prior to use. Data were recorded with Astra software (Wyatt; version 7.1.4.8).

For the SEC-MS experiments, a T-joint was inserted before the RI detector to prevent overpressure in the cell compartment, and a PEEK tubing of about 70 cm was used to transfer the samples into an LTQ Orbitrap Velos (Thermo Fisher Scientific, Waltham, MA, USA), with an APCI source operating in positive ionization mode. The resolution was set to 30.000, and calibration was performed using external standards. Spectra were collected from 80–1.000 *m*/*z* and data were analyzed using Xcalibur (Thermo Fisher Scientific; version 2.2 SP1.48).

#### 3.3.3. HPLC-MS

Analyses were performed by reversed-phase chromatography using a Surveyor HPLC (Thermo Fisher Scientific) equipped with a Zorbax SB-C18 column (150 mm × 2.1 mm, 5 μm; Agilent). The column temperature was set to 40 °C, and the injection volume was 1 μL. Analytes were separated by gradient elution with mobile phase A containing 0.1% formic acid (FA) in water and mobile phase B containing 0.1% FA in acetonitrile at a flow rate of 0.2 mL min^−1^. The elution gradient starting conditions were 90% A and 10% B. After 1 min, the proportion of B was increased to 95% at 25 min, where it was held for further 35 min. High-resolution mass spectra were obtained using an LTQ Orbitrap Velios (Thermo Fisher Scientific), with an APCI source operated in positive ionization mode. The resolution was set to 30.000, and diisooctylphthalate (*m*/*z* = 391.2843) was used as internal standard for mass calibration. Spectra were collected from 80–1.000 *m*/*z*, and data were analyzed using Xcalibur (Thermo Fisher Scientific; version 2.2 SP1.48).

#### 3.3.4. FTIR

Spectra were collected with a diamond ATR unit on an iZ10 bench attached to an iN10 MX FTIR microscope (Thermo Fisher Scientific). Resolution was set to 4 cm^−1^, spectral range from 600–4000 cm^−1^, and 32 spectra were collected and averaged, except for the experiments under argon, where only 8 scans were used.

## 4. Conclusions

Various BIANs have successfully been used to reduce the viscosity of alkyl magnesium solutions in heptane and toluene. While, in part, they react with the alkyl groups and form stable products, the mechanism of viscosity reduction is assigned to the formation of a complex between magnesium and the C = N bonds of the BIANs, which interferes with the polymeric structure of the alkyl magnesium in the solution, as could be shown with inert FTIR and NMR measurements. This becomes evident as, especially, the *i*-Pr-BIAN, respectively, its butylated derivative, can be reused for viscosity reduction several times, and only after the first use is an alkylated moiety formed. In all further experiments, no more change in the chemical structure, e.g., by alkylation, was observed; thus, it is unlikely that alkyl transfer is the sole cause of the reduced viscosity. The influence of substituents on the BIAN structure has been determined to be almost inexistant, in terms of viscosity reduction behavior; however, the chemical stability of the reaction products is strongly influenced by side groups. While the reaction products of BIAN, F_3_C-BIAN, and MeO-BIAN might influence the further use of the alkyl magnesium solutions, i-Pr-BIAN has the lowest risk of doing so and, thus, is found to be the best candidate for further use.

In order to better understand how type and position of substituents influence the chemical stability, further experiments with additional BIANs and analogous compounds must be performed to elucidate the exact mechanism involved.

## Figures and Tables

**Table 1:** Viscosity reduction effect and color change by the reaction of BOMAG (19.3% in toluene or 20.4% in heptane), with the respective BIANs.

Additive	Solvent	*η* (mPa s)	*w*_mol_ (mol%)	Color _BIAN_ *	Color _Reaction Solution_
-	toluene	66.7	-	-	colorless
-	heptane	40.2	-	-	colorless
BIAN	toluene	15.7	2.54	yellow	dark purple
BIAN	heptane	11.3	2.30	yellow	red
*i*-Pr-BIAN	toluene	23.2	2.45	orange	purple
*i*-Pr-BIAN	heptane	13.4	2.21	yellow/orange	purple
MeO-BIAN	toluene	23.2	2.48	red	dark red
MeO-BIAN	heptane	12.1	2.55	red	violet/red
F_3_C-BIAN	toluene	34.1	2.53	orange	dark red
F_3_C-BIAN	heptane	25.3	2.13	yellow	dark red

* In solution.

**Table 2:** MS identification of the active BIAN—alkyl magnesium compounds.

Structure	Alkyl Residue	*t*_R_/min	*m*/*z* _Observed_	*m*/*z* _Calculated_
BIAN	-	13.82	333.1382	333.1386
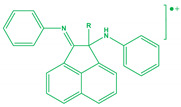	ethyl	9.61	362.1781	362.1778
	butyl	9.11	390.2093	390.2091
	octyl	8.44	446.2717	446.2717
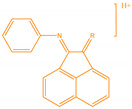	ethyl	11.93	270.1279	270.1277
	butyl	10.72	298.1592	298.1590
	octyl	9.48	354.2218	354.2216

**Table 3:** HPLC-MS results of quenched BIANs reacted with dibutyl magnesium.

Educt	Entry	*t*_R_/min	Structure *	*m*/*z* _Observed_	*m*/*z* _Calculated_
*i*-Pr-BIAN	1	36.56	*i*-Pr-BIAN	501.3260	501.3264
	2	48.28	butylated BIAN with double bond	557.3887	557.3890
	3	51.54	butylated BIAN	559.4045	559.4047
MeO-BIAN	4	2.03	methoxy aniline	124.0757	124.0757
	5	9.02	*N*-butyl methoxy aniline	180.1380	180.1383
	6	14.92	acenaphtoquinone	183.0439	183.0441
	7	15.38	unknown	199.0388	
	8	18.64	unknown	304.0965	
	9	22.49	unknown	304.0965	
	10	23.25	unknown	304.0965	
	11	24.95	doubly butylated monoketone	402.2426	402.2428
	12	26.15	butylated MeO-BIAN	451.2378	451.2380
	13	29.81	doubly butylated MeO-BIAN	507.3004	507.3006
	14	30.30	unknown	427.2741	
	15	31.79	doubly butylated MeO-BIAN	507.3007	507.3006
	16	35.44	doubly butylated MeO-BIAN with double bond	505.2848	505.2850
F_3_C-BIAN	6	14.90	acenaphtoquinone	183.0439	183.0441
	17	15.35	unknown	199.0387	
	18	18.81	butylated acenaphtoquinone	223.1115	223.1117
	19	21.28	unknown	416.1466	
	20	23.98	*N*-butyl trifluoromethylaniline	218.1149	218.1151
	21	24.60	unknown	342.0735	
	22	25.48	butylated monoketone	384.1568	384.1570
	23	28.36	butylated butyl half-BIAN	424.2245	424.2247
	24	29.68	doubly butylated monoketone	440.2193	440.2196
	25	31.29	butylated F_3_C-BIAN	527.1913	527.1916
	26	31.90	unknown	359.3154	
BIAN	27	2.5	aniline	94.0652	94.0651
	28	11.20	*N*-butyl aniline	150.1276	150.1277
	6	15.10	acenaphthoquinone	183.0439	183.0441
	29	18.84	unknown	274.0860	
	30	22.68	unknown	274.0859	
	31	24.03	butylated monoketone	316.1692	316.1696
	32	24.67	unknown	330.1487	
	33	27.88	doubly butylated monoketone	372.2322	372.2322
	34	28.25	butylated BIAN	391.2172	391.2169
	35	31.87	doubly butylated BIAN (no more double bond left)	449.2950	449.2951
	36	32.70	double butylated BIAN with double bond	447.2794	447.2795
	37	35.15	isomer of 36 (*N*-butylated)	447.2793	447.2795

* Structures can be found in the Supplementary Materials Scheme S1.

**Table 4:** Viscosity results for the pure BIAN substances and reused quenched products with BOMAG in toluene.

Additive	Extraction Number	*η* (mPa s)	*m*_Modifier_ (mg)	*m*_BOMAG_ (mg)	Color_Reaction Solution_	Color_Extracted Solid_
-	-	66.7	-	-	-	
*i*-Pr-BIAN	1	17.2	150.4	7.153	purple	brown
	2	28.4	119.0	7.411	purple	light brown
MeO-BIAN	1	44.5	97.9	7.351	red	orange/yellow
F_3_C-BIAN	1	42.6	107.0	7.473	red	orange/yellow

## Data Availability

Not applicable.

## Supplementary Materials

The following supporting information can be downloaded at: https://www.mdpi.com/article/10.3390/molecules28020489/s1, Figure S1: 1H-NMR of BIAN in CDCl3; Figure S2: 13C-NMR of BIAN in CDCl3; Figure S3: 1H-NMR of i-Pr-BIAN in CDCl3; Figure S4: 13C-NMR of i-Pr-BIAN in CDCl3; Figure S5: 1H-NMR of MeO-BIAN in CDCl3; Figure S6: 13C-NMR of MeO-BIAN in CDCl3; Figure S7: 1H-NMR of F3C-BIAN in CDCl3; Figure S8: Size exclusion chromatograms using UV (top) and RI (bottom) detection: (a) analysis of F3C-BIAN (black), BEM (red), and the mixture of BEM with F3C-BIAN (green) and (b) F3C-BIAN (black), BOMAG (red), and the mixture of BOMAG with F3C-BIAN (green); Figure S9: SEC-MS results of F3C-BIAN. In black pure F3C-BIAN with *m*/*z* = 468.1061, and furthermore the radical structures formed with F3C-BIAN with BOMAG and BEM at the following *m*/*z* ratios: 582.2481 (red), 526.1847 (green and blue) and 498.1534 (yellow); Figure S10: SEC-MS results of F3C-BIAN. In black pure F3C-BIAN with *m*/*z* = 468.1061, and furthermore the double bound structures formed with F3C-BIAN with BOMAG and BEM at the following m/z ratios: 422.2105 (red), 366.1470 (green and blue) and 338.1158 (yellow); Figure S11: Size exclusion chromatograms using UV (top) and RI (bottom) detection: (a) analysis of MeO-BIAN (black), BEM (red), and the mixture of BEM with MeO-BIAN (green) and (b) MeO-BIAN (black), BOMAG (red), and the mixture of BOMAG with MeO-BIAN (green); Figure S12: SEC-MS results of MeO-BIAN. In black pure MeO-BIAN with *m*/*z* = 393.1597, and furthermore the radical structures formed with MeO-BIAN with BOMAG and BEM at the following *m*/*z* ratios: 506.2931 (red), 450.2314 (green and blue) and 422.1999 (yellow); Figure S13: SEC-MS results of MeO-BIAN. In black pure MeO-BIAN with *m*/*z* = 393.1597, and furthermore the double bound structures formed with MeO-BIAN with BOMAG and BEM at the following *m*/*z* ratios: 384.2326 (red), 328.1698 (green and blue) and 300.1389 (yellow); Figure S14: Size exclusion chromatograms using UV (top) and RI (bottom) detection: analysis of i-Pr--BIAN (black), BOMAG (red), and the mixture of BOMAG with i-Pr--BIAN (green); Figure S15: SEC-MS results of i-Pr-BIAN. In black pure i-Pr-BIAN with *m*/*z* = 501.3268 and furthermore the structures formed with i-Pr-BIAN with BOMAG and BEM at the following *m*/*z* ratios: 615.4688 (red), 559.4050 (green and blue) and 531.3739 (yellow); Figure S16: 1H-NMR of i-Pr-BIAN modified BOMAG in D6C6; Figure S17: 13C-NMR of i-Pr-BIAN modified BOMAG in D6C6; Figure S18: 1H-NMR of quenched MeO-BIAN in CDCl3; Figure S19: 13C-NMR of quenched MeO-BIAN in CDCl3; Figure S20: 1H-NMR of quenched F3C-BIAN in CDCl3; Figure S21: 13C-NMR of quenched F3C-BIAN in CDCl3; Scheme S1: Tentative structures for the quenched BIAN products with dibutyl magnesium; Table S1: MS identification of the active F3C-BIAN—alkyl magnesium compounds; Table S1: MS iden-tification of the active MeO-BIAN—alkyl magnesium compounds; Table S2: MS identification of the active i-Pr-BIAN—alkyl magnesium compounds.

## Sample Availability

Samples of the compounds are available from the authors.

## References

[1] Fannin L.W., Malpass D.B., Sanchez R. (1979). Hydrocarbon Soluble Magnesium Compositions of High Magnesium Content.

[2] Fannin L.W., Malpass D.B., Sanchez R. (1980). Organomagnesium Solutions of Low Viscosity.

[3] Kobetz P., Pinkerton R.C. (1962). Manufacture of Magnesium Organo Compounds.

[4] Malpass D.B., Fannin L.W., Sanchez R. (1984). Hydrocarbon Soluble Dialkylmagnesium Composition.

[5] Teruhiko I., Naoshi M., Seiichiro M., Kiyoshi S. (1964). Process for the Production of a High Molecular Weight Polymer of an Epoxide Using a Three Component Catalyst Comprising an Organozinc Compound and Organomagnesium Compound and Water.

[6] Kamienski C.U., Eastham J.U. (1970). Method of Preparing Telomers Utilizing as Catalysts Hydrocarbon Soluble Organometallic Complexes of Metals of Groups I and IIA of the Periodic Table.

[7] Kamienski C.W., Mcelroy B.J., Bach R.O. (1976). Stable Diorganomagnesium Compositions.

[8] Conrad W.K., Jerome F.E. (1970). Diorganomagnesium Reagents and Methods of Preparing Same.

[9] Fannin L.W., Malpass D.B. (1977). Hydrocarbon Soluble Straight-Chain Di-(Lower Alkyl) Magnesium Compositions.

[10] Galli P., Vecellio G. (2001). Technology: Driving force behind innovation and growth of polyolefins. Prog. Polym. Sci..

[11] Soares J.B.P., McKenna T.F.L. (2012). Polyolefin Reaction Engineering.

[12] Albunia A.R., Prades F., Jeremic D. (2019). Multimodal Polymers with Supported Catalysts.

[13] Severn J.R., Albunia A.R., Prades F., Jeremic D. (2019). Recent Developments in Supported Polyolefin Catalysts: A Review. Multimodal Polymers with Supported Catalysts.

[14] (2000). Ullmann’s Encyclopedia of Industrial Chemistry.

[15] Malpass D.B. (2012). Introduction to Industrial Polypropylene: Properties, Catalysts, Processes.

[16] Aigner P., Averina E., Garoff T., Paulik C. (2017). Effects of Alterations to Ziegler-Natta Catalysts on Kinetics and Comonomer (1-Butene) Incorporation. Macromol. React. Eng..

[17] Garoff T., Waldvogel P., Personen K., Waldvogel P., Pesonen K. (2005). Method for the Preparation of Olefin Polymerization Catalyst Support and an Olefin Polymerization Catalyst.

[18] Rönkkö H.-L., Knuuttila H., Denifl P., Leinonen T., Venäläinen T. (2007). Structural studies on a solid self-supported Ziegler–Natta-type catalyst for propylene polymerization. J. Mol. Catal. A Chem..

[19] Leinonen T., Denifl P. (2001). Preparation of Olefin Polymerisation Catalyst Component.

[20] Hoff R.E., Handbook of Transition Metal Polymerization Catalysts (2010).

[21] Screttas C.G., Micha-Screttas M. (1985). Preparation of solvated and/or unsolvated simple and mixed diarylmagnesiums. J. Organomet. Chem..

[22] Stuhl C., Anwander R. (2018). Dimethylmagnesium revisited. Dalton Trans..

[23] Weiss E. (1964). Die kristallstruktur des dimethylmagnesiums. J. Organomet. Chem..

[24] Saulys D.A., Hill E.A., Scott R.A. (2011). Beryllium & Magnesium: Organometallic Chemistry. Encyclopedia of Inorganic and Bioinorganic Chemistry.

[25] Malpass D.B., Webb D.W. (1984). Organomagnesium Solutions of Low Viscosity.

[26] De Vries M.N. (1970). Process for the Preparation of Dialkyl Magnesium Compound.

[27] Jones P.D., Malpass D.B., Smith G.M. (1997). Viscosity Reduction of Organomagnesium Solutions.

[28] Kamienski C.W., Dover B.T. (1989). Low Viscosity Hydrocarbon Solution of Dialkylmagnesium Compounds.

[29] Schwarz J.F., Holtrichter-Rößmann T., Liedtke C.G., Diddens D., Paulik C. (2022). Modified Magnesium Alkyls for Ziegler–Natta Catalysts. Catalysts.

[30] Holtrichter R.T., Liedke C., Schwarz J. (2021). Novel Organo-Magnesium Compounds and Their Use.

[31] Chlupatý T., Bílek M., Merna J., Brus J., Růzičková Z., Strassner T., Růzička A. (2019). The addition of Grignard reagents to carbodiimides. The synthesis, structure and potential utilization of magnesium amidinates. Dalton Trans..

[32] Anga S., Bhattacharjee J., Harinath A., Panda T.K. (2015). Synthesis, structure and reactivity study of magnesium amidinato complexes derived from carbodiimides and N,N’-bis(2,6-diisopropylphenyl)-1,4-diaza-butadiene ligands. Dalton Trans..

[33] Joshi A., Collins S., Linnolahti M., Zijlstra H.S., Liles E., McIndoe J.S. (2021). Spectroscopic studies of synthetic methylaluminoxane: Structure of methylaluminoxane activators. Chemistry.

[34] Joshi A., Killeen C., Thiessen T., Zijlstra H.S., McIndoe J.S. (2022). Handling considerations for the mass spectrometry of reactive organometallic compounds. J. Mass Spectrom..

[35] (2021). Samuel Redl. Extending the Range of Ar-BIAN-Supported N-Heterocyclic Carbene Complexes and Their Catalytic Application in Hydrogenation Reactions. Master’s Thesis.

[36] (2019). Daniel Timelthaler. Development of Base Metal Catalysts for Green Hydrogenation Reactions. Master’s Thesis.

[37] van Asselt R., Elsevier C.J., Smeets W.J.J., Spek A.L., Benedix R. (1994). Synthesis and characterization of rigid bidentate nitrogen ligands and some examples of coordination to divalent palladium. X-ray crystal structures of bis (p-tolylimino) acenaphthene and methylchloro [bis(o,o′-diisopropylphenyl-imino) acenaphthene] palla. Recl. Trav. Chim. Pays-Bas.

[38] Gasperini M., Ragaini F., Cenini S. (2002). Synthesis of Ar-BIAN Ligands (Ar-BIAN = Bis(aryl)acenaphthenequinonediimine) Having Strong Electron-Withdrawing Substituents on the Aryl Rings and Their Relative Coordination Strength toward Palladium(0) and -(II) Complexes. Organometallics.

[39] Infrared Spectroscopy Committee of the Chicago Society for Coatings Technology, Federation of Societies for Coatings Technology (1980). An Infrared Spectroscopy Atlas for the Coatings Industry.

[40] IR-Spektrum: Tabelle & Schema. https://www.sigmaaldrich.com/AT/de/technical-documents/technical-article/analytical-chemistry/photometry-and-reflectometry/ir-spectrum-table.

[41] Gaballa A.S., Asker M.S., Barakat A.S., Teleb S.M. (2007). Synthesis, characterization and biological activity of some platinum(II) complexes with Schiff bases derived from salicylaldehyde, 2-furaldehyde and phenylenediamine. Spectrochim. Acta A Mol. Biomol. Spectrosc..

[42] Rosa V., Avilés T., Aullon G., Covelo B., Lodeiro C. (2008). A new bis(1-naphthylimino)acenaphthene compound and its Pd(II) and Zn(II) complexes: Synthesis, characterization, solid-state structures and density functional theory studies on the syn and anti isomers. Inorg. Chem..

[43] Aranha P.E., dos Santos M.P., Romera S., Dockal E.R. (2007). Synthesis, characterization, and spectroscopic studies of tetradentate Schiff base chromium(III) complexes. Polyhedron.

[44] Caro C.A., Cabello G., Landaeta E., Pérez J., González M., Zagal J.H., Lillo L. (2014). Preparation, spectroscopic, and electrochemical characterization of metal(II) complexes with Schiff base ligands derived from chitosan: Correlations of redox potentials with Hammett parameters. J. Coord. Chem..

