# Supplementary material for: Structural Characterization of BIAN Type Molecules Used in Viscosity Reduction of Alkyl Magnesium Solutions
Source: Molecules. 2023 Jan 4;28(2):489. doi: 10.3390/molecules28020489 (PMC9865295; doi:10.3390/molecules28020489)
Supplement: Supplementary file 1 [file molecules-28-00489-s001.zip › molecules-2101616-supplementary.pdf]

molecules

Supporting Information

Structural Characterization of BIAN type molecules used in viscosity reduction of alkyl magnesium solutions

Julia Felicitas Schwarz [1], Lukas Gadermayr [1], Samuel Redl[2], Thorsten Holtrichter-Rößmann[3], Christian Paulik[1] and Clemens Schwarzinger [1,*]

[1] Institute for Chemical Technology of Organic Materials, Johannes Kepler University (JKU), Altenberger Strasse 69, 4040 Linz, Austria
[2] Institute of Inorganic Chemistry, Johannes Kepler University (JKU), Altenberger Strasse 69, 4040 Linz, Austria
[3] Lanxess Organometallics GmbH, Ernst-Schering-Straße 14, 59192 Bergkamen, Germany
* Correspondence: clemens.schwarzinger@jku.at

1. NMR spectroscopy of BIAN derivatives

a. ***N*[1],*N*[2]-Diphenylacenaphthylene-1,2-diimine (BIAN):** Yellow crystalline solid

Analytical data:

^{1}H NMR (300 MHz, $CDCl_3$, 20 °C): δ = 7.91 (d, *J* = 8.28 Hz, 2H), 7.50 (t, *J* = 7.50 Hz, 4H), 7.39 (t, *J* = 7.76 Hz, 2H), 7.29 (t, *J* = 7.37 Hz, 3H, overlapping), 7.16 (d, *J* = 7.83 Hz, 4H), 6.86 (d, *J* = 7.23 Hz, 2H) ppm;

^{13}C NMR (75.5 MHz, $CDCl_3$, 20 °C): δ = 161.3, 151.8, 141.8, 131.2, 129.4, 129.0, 128.5, 127.7, 124.4, 124.0, 118.2 ppm;

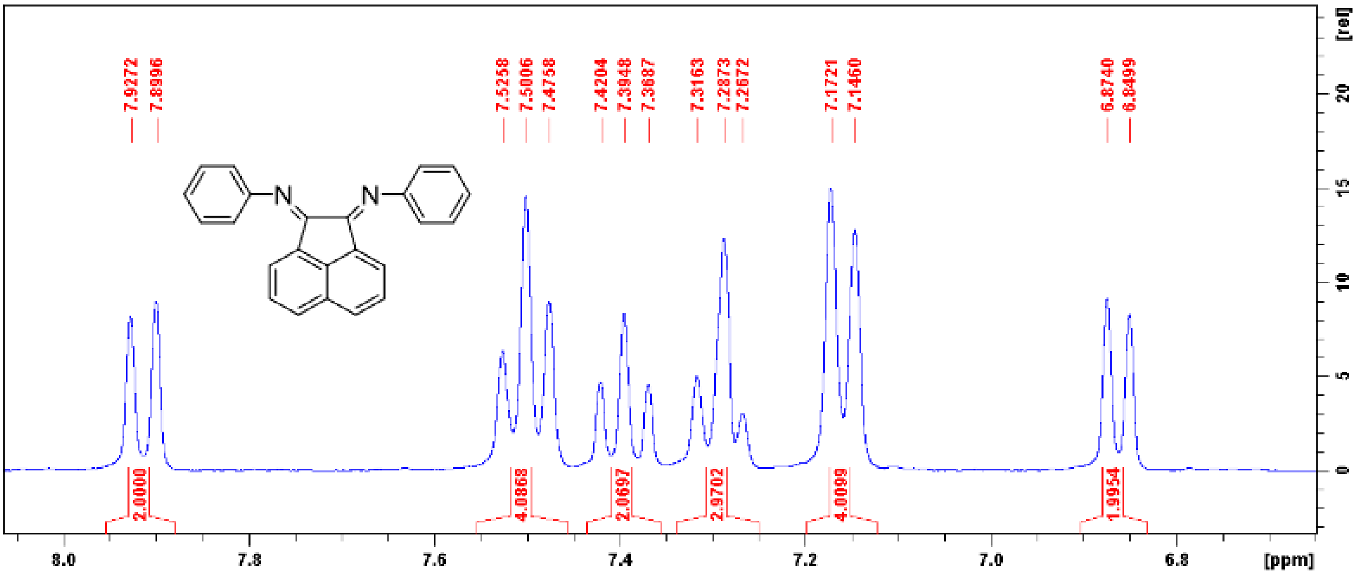

Figure S1. ^{1}H-NMR of BIAN in $CDCl_3$.

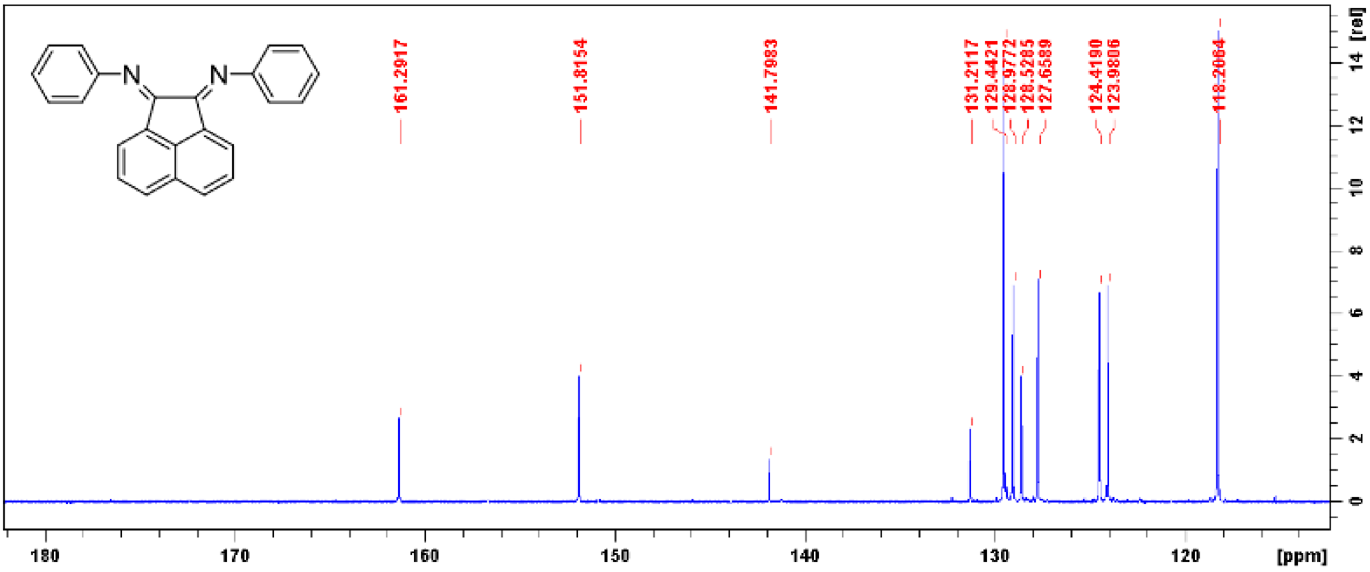

Figure S2. ^{13}C-NMR of BIAN in $CDCl_3$.

b. ***N***

b. ***N*¹,*N*²-Bis(2,6-diisopropylphenyl)acenaphthylene-1,2-diimine (*i*-Pr-BIAN):** Orange crystalline solid

Analytical data:

^{1}H NMR (300 MHz, $CDCl_3$, 20 °C): δ = 7.86 (d, *J* = 8.28 Hz, 2H), 7.36 (t, *J* = 7.77 Hz, 2H), 7.30 – 7.20 (m, 6H, overlapping), 6.65 (d, *J* = 7.14 Hz, 2H), 2.96 – 3.10 (m, 4H), 1.23 (d, J = 6.84 Hz, 12H), 0.98 (d, *J* = 6.78 Hz, 12H) ppm;

^{13}C NMR (75.5 MHz, $CDCl_3$, 20 °C): δ = 161.0, 147.5, 135.4, 131.1, 129.5, 128.9, 127.9, 124.3, 123.5, 123.4, 28.6, 23.6, 23.1 ppm.

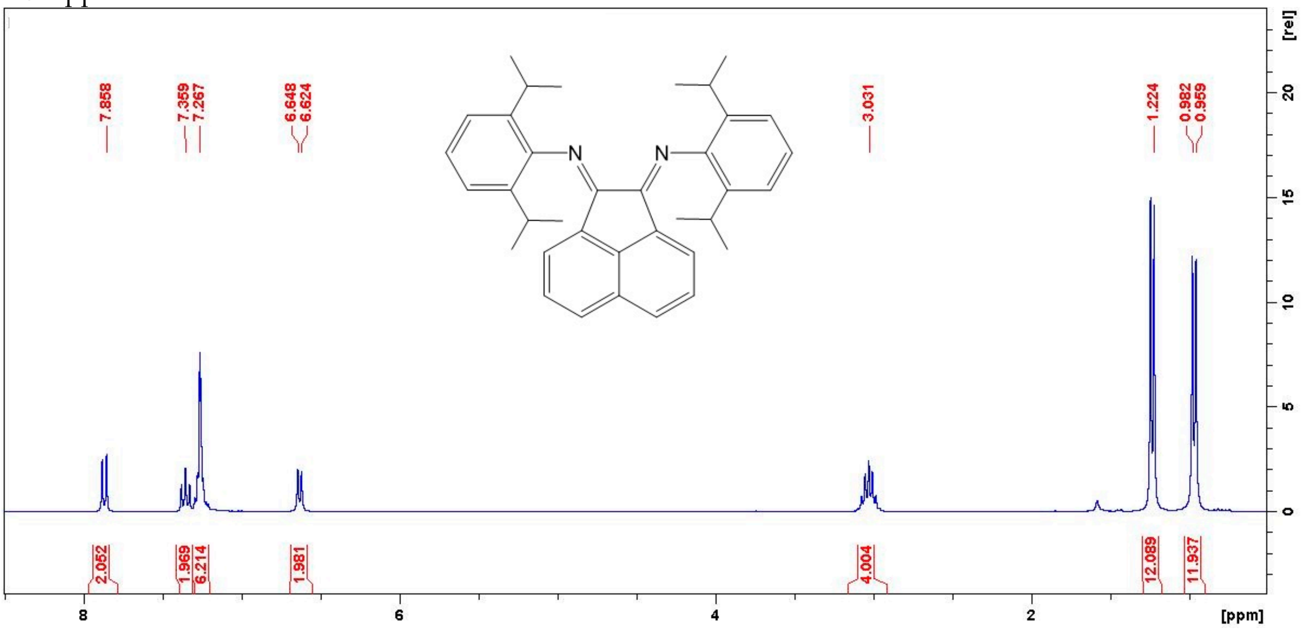

Figure S3. ^{1}H-NMR of *i*-Pr-BIAN in $CDCl_3$.

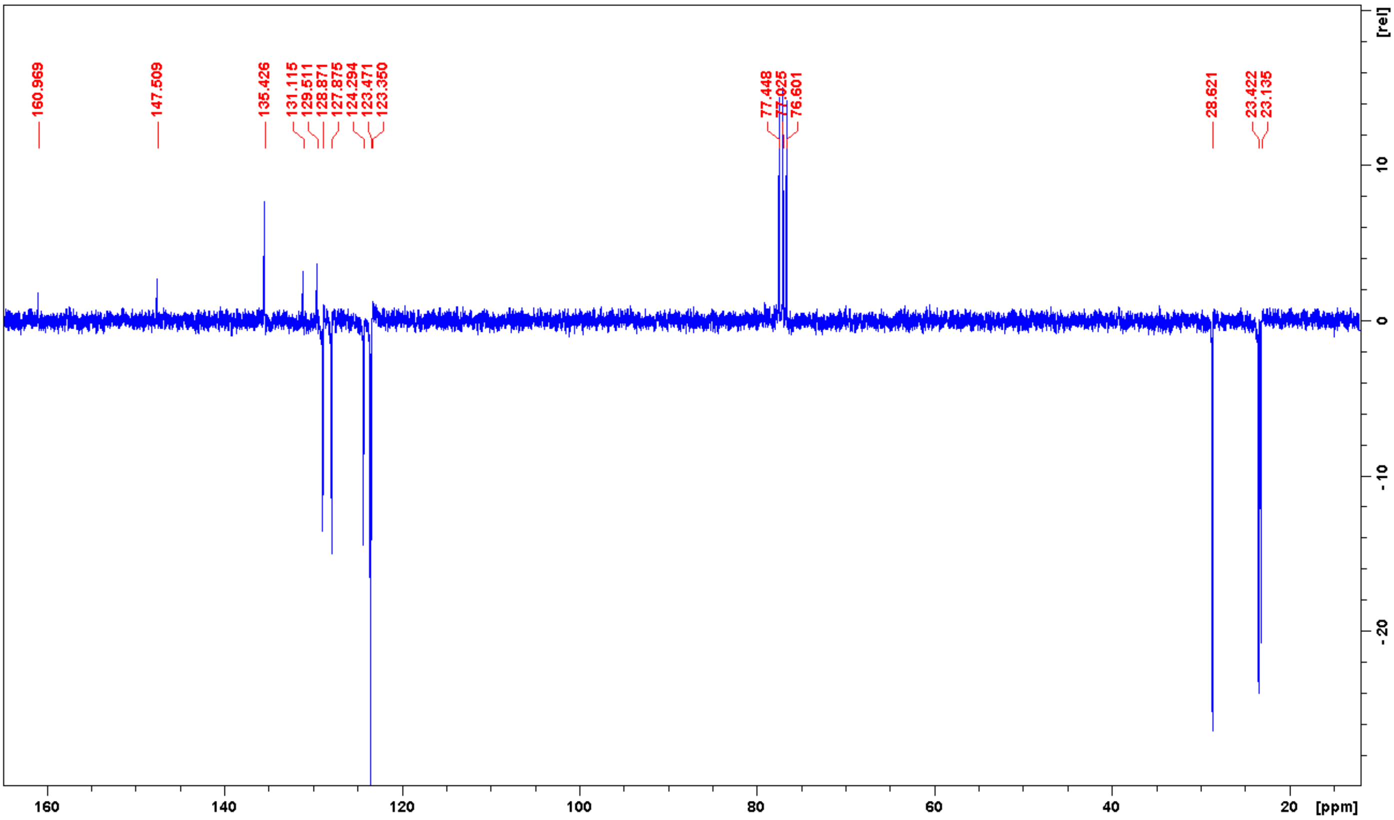

Figure S4. ^{13}C-NMR of *i*-Pr-BIAN in $CDCl_3$

c. ***N*¹,*N*²-Bis(4-methoxyphenyl)acenaphthylene-1,2-diimine (MeO-BIAN):** dark red crystalline solid
Analytical data:
^{1}H NMR (300 MHz, $CDCl_3$, 20 °C): δ = 7.89 (d, *J* = 8.28 Hz, 2H), 7.39 (t, *J* = 7.74, 2H), 7.11 – 7.09 (m, 4H), 7.05 – 7.00 (m, 6H), 3.81 (s, 6H) ppm;

^{13}C NMR (75.5 MHz, $CDCl_3$, 20 °C): δ = 161.7, 157.0, 145.0, 141.8, 131.4, 129.0, 128.9, 127.7, 123.8, 119.9, 114.8, 55.6 ppm.

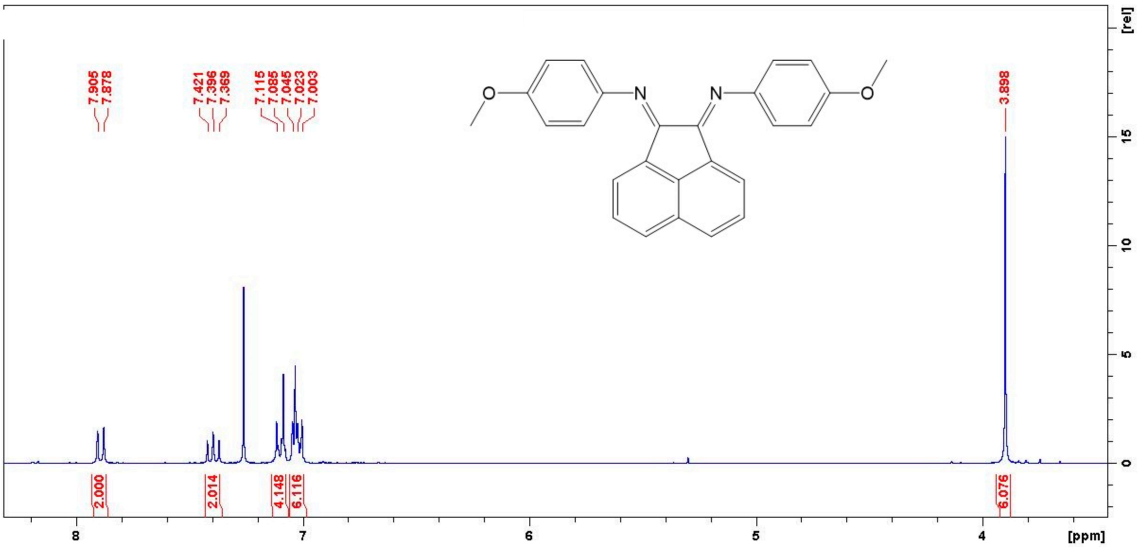

Figure S5. ^{1}H-NMR of MeO-BIAN in $CDCl_3$.

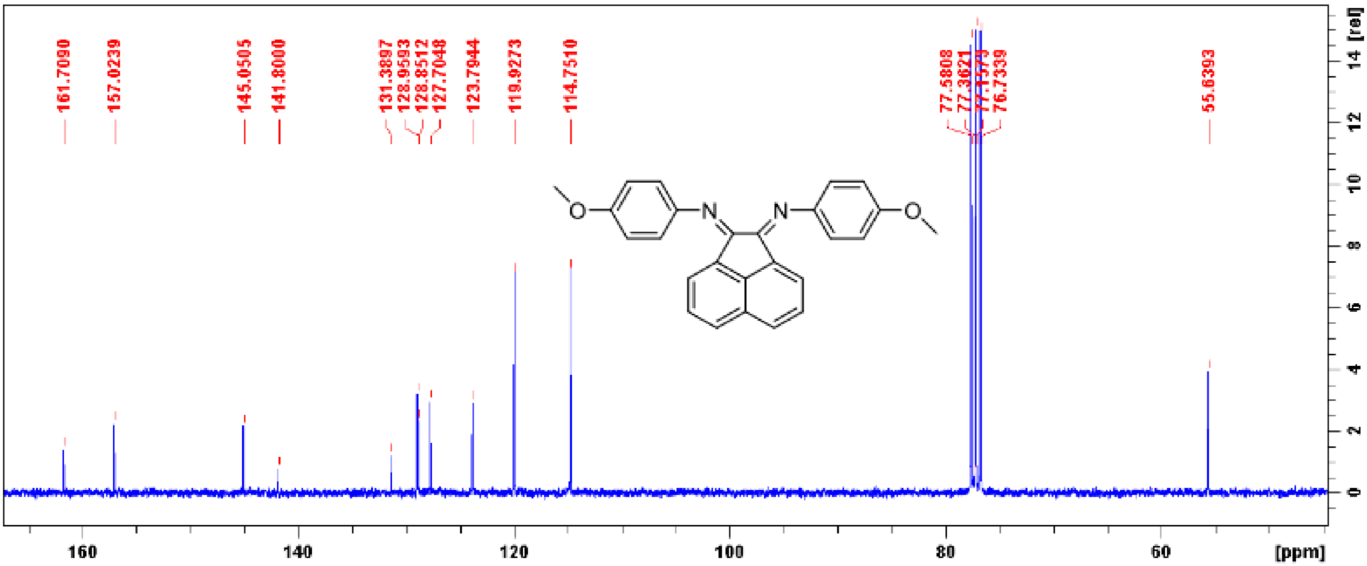

Figure S6. ^{13}C-NMR of MeO-BIAN in $CDCl_3$.

d. ***N*[1],*N*[2]-Bis(3-(trifluoromethyl)phenyl)acenaphthylene-1,2-diimine (F_3C-BIAN):** yellow crystalline solid

Analytical data:

^{1}H NMR (300 MHz, $CDCl_3$, 20 °C): δ = 8.0 (d, *J* = 8.19 Hz, 2H), 7.61 – 7.57 (m, 4H), 7.49 – 7.44 (m, 4H), 7.30 (d, *J* = 7.8 Hz, 2H), 6.90 (d, J = 7.0 Hz, 2H) ppm;

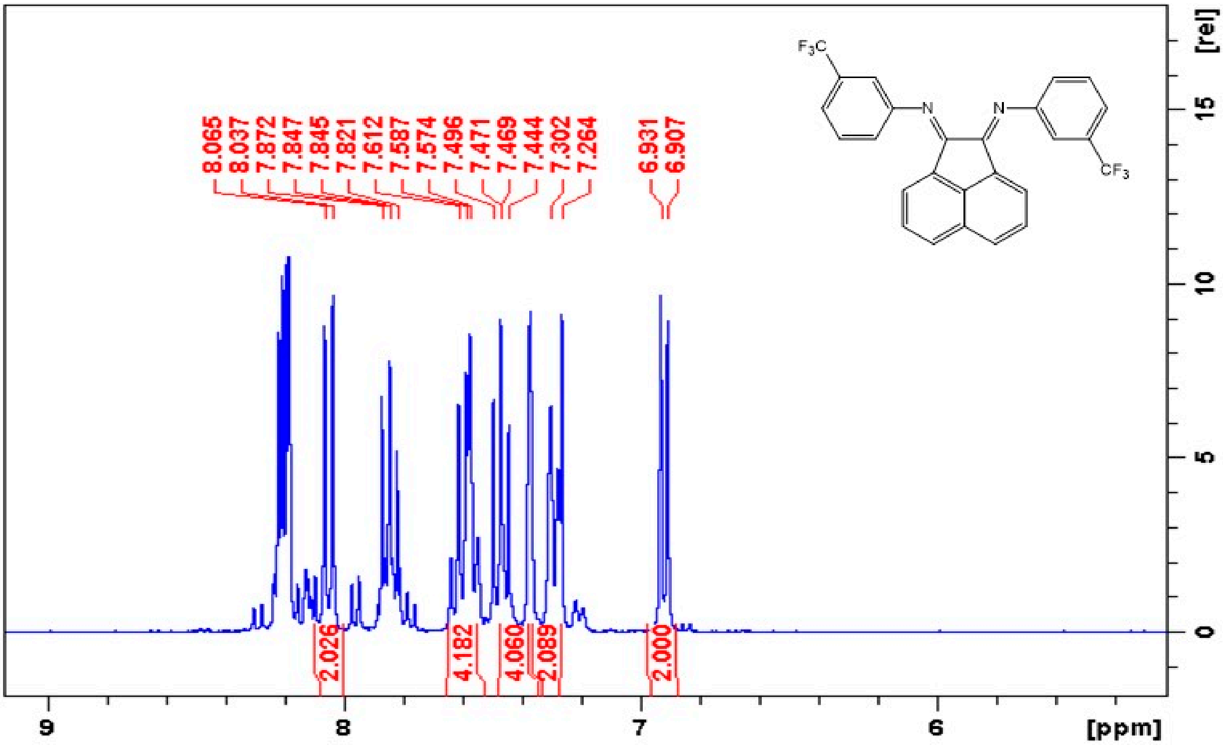

Figure S7. ^{1}H-NMR of F_3C-BIAN in $CDCl_3$.

2. SEC-MS Experiments

a. F_3C-BIAN

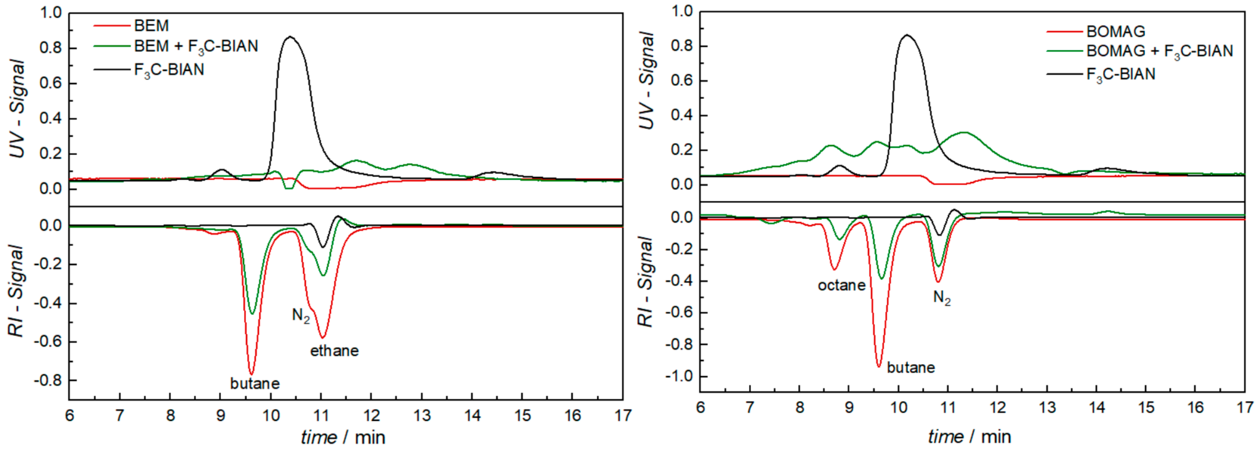

(**a**) (**b**)

Figure S8. Size exclusion chromatograms using UV (top) and RI (bottom) detection: (a) analysis of F_3C-BIAN (black), BEM (red), and the mixture of BEM with F_3C-BIAN (green) and (b) F_3C-BIAN (black), BOMAG (red), and the mixture of BOMAG with F_3C-BIAN (green).

Table S1. MS identification of the active F_3C-BIAN – alkyl magnesium compounds.

Structure	Alkyl residue -R	t_R / min	m/z observed	m/z calculated
F_3C-BIAN	-	8.80	469.1133	468.1061
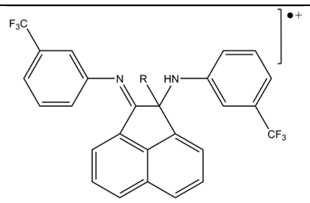	ethyl	9.36	498.1534	498.1525
	butyl	8.90	526.1847	526.1838
	octyl	8.30	582.2481	582.2464
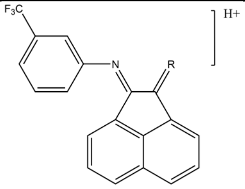	ethyl	13.23	338.1158	338.1151
	butyl	12.10	366.1470	366.1464
	octyl	10.27	422.2105	422.2090

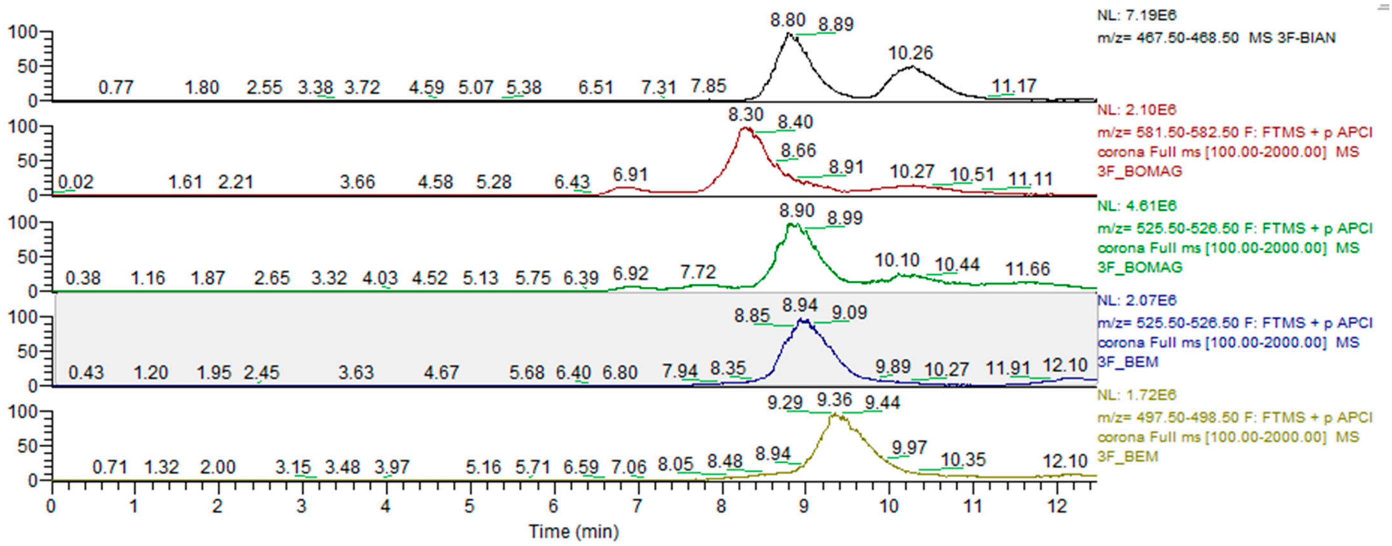

Figure S9. SEC-MS results of F_3C-BIAN. In black pure F_3C-BIAN with m/z= 468.1061, and furthermore the radical structures formed with F_3C-BIAN with BOMAG and BEM at the following m/z ratios: 582.2481 (red), 526.1847 (green and blue) and 498.1534 (yellow).

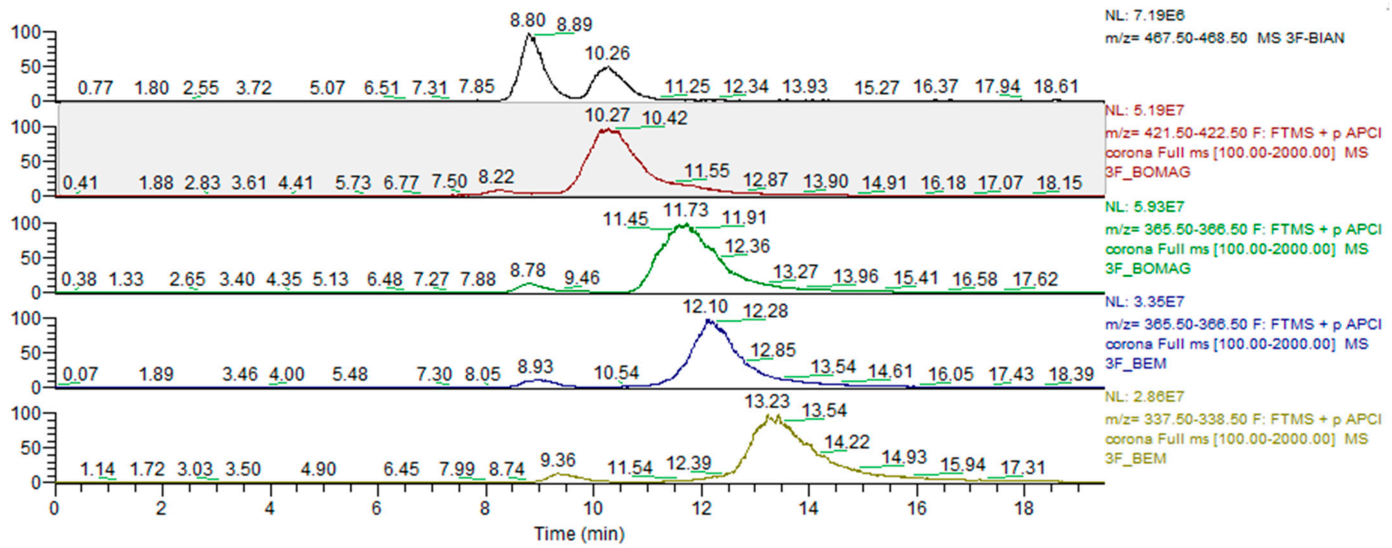

Figure S10. SEC-MS results of F_3C-BIAN. In black pure F_3C-BIAN with m/z= 468.1061, and furthermore the double bound structures formed with F_3C-BIAN with BOMAG and BEM at the following m/z ratios: 422.2105 (red), 366.1470 (green and blue) and 338.1158 (yellow).

b. MeO-BIAN

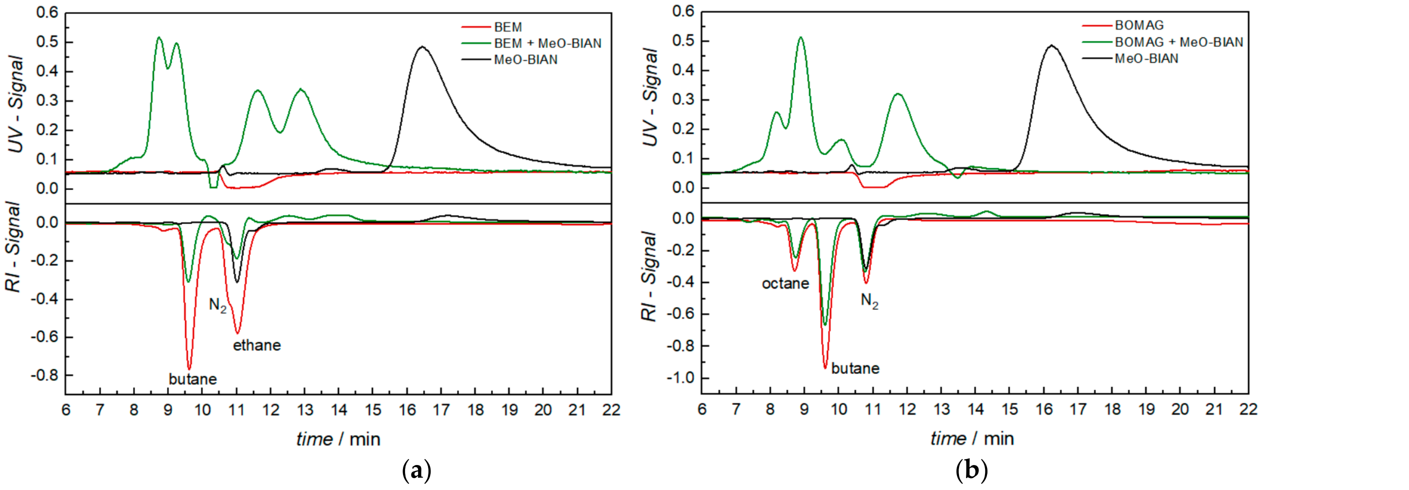

Figure S11. Size exclusion chromatograms using UV (top) and RI (bottom) detection: (a) analysis of MeO-BIAN (black), BEM (red), and the mixture of BEM with MeO-BIAN (green) and (b) MeO-BIAN (black), BOMAG (red), and the mixture of BOMAG with MeO-BIAN (green).

Table S2. MS identification of the active MeO-BIAN – alkyl magnesium compounds.

Structure	Alkyl residue - R	t_R / min	m/z observed	m/z calculated
MeO-BIAN	-	16.42	393.1597	393.1603
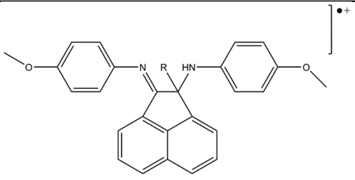	ethyl	9.63	422.1999	422.1989
	butyl	9.10	450.2314	450.2302
	octyl	9.46	506.2931	506.2928
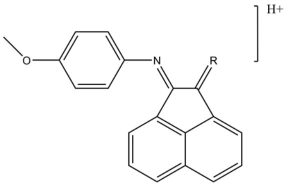	ethyl	13.25	300.1389	300.1383
	butyl	12.01	328.1698	328.1696
	octyl	11.55	384.2326	384.2322

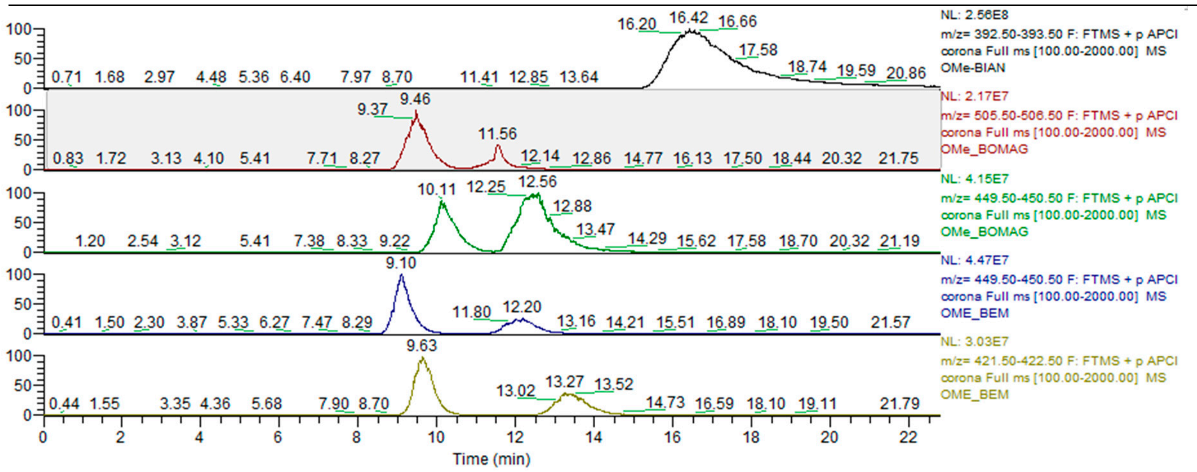

Figure S12. SEC-MS results of MeO-BIAN. In black pure MeO-BIAN with m/z= 393.1597, and furthermore the radical structures formed with MeO-BIAN with BOMAG and BEM at the following m/z ratios: 506.2931 (red), 450.2314 (green and blue) and 422.1999 (yellow).

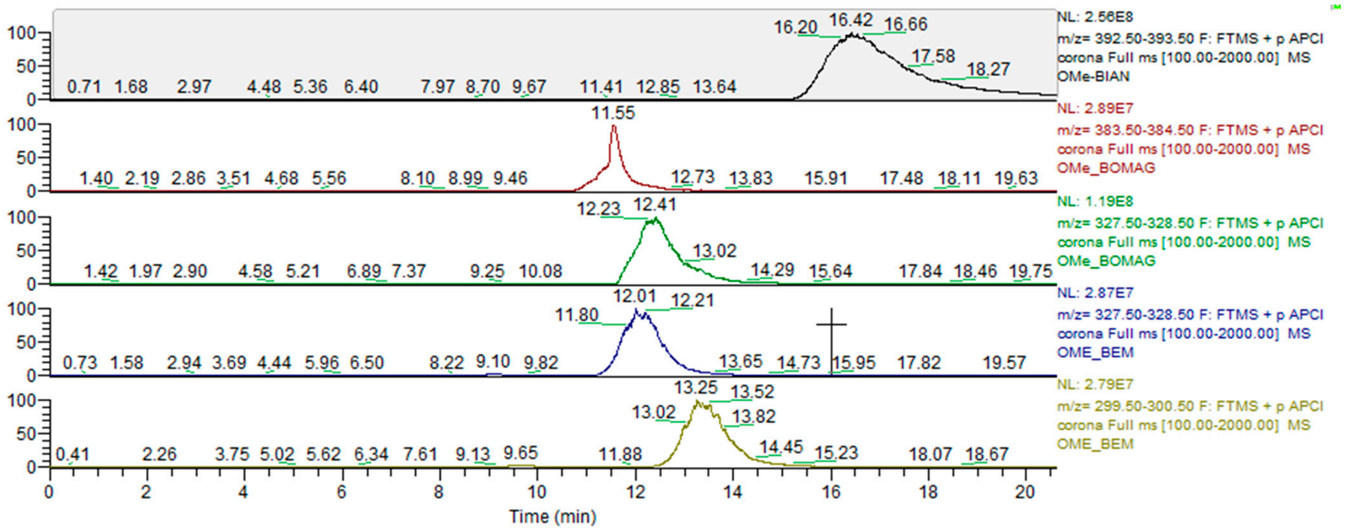

Figure S 13. SEC-MS results of MeO-BIAN. In black pure MeO-BIAN with m/z= 393.1597, and furthermore the double bound structures formed with MeO-BIAN with BOMAG and BEM at the following m/z ratios: 384.2326 (red), 328.1698 (green and blue) and 300.1389 (yellow).

c. *i*-Pr-BIAN

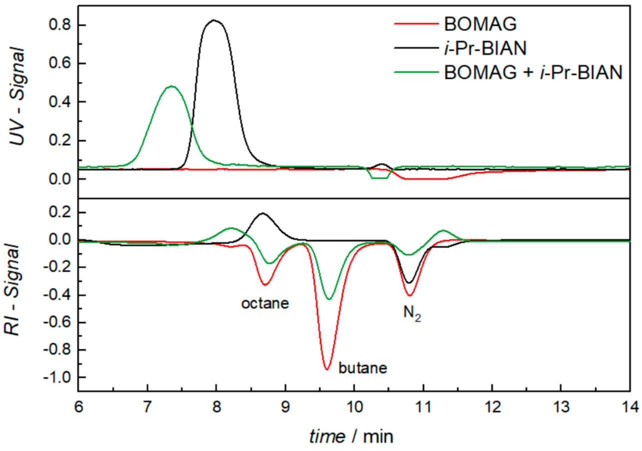

Figure S14. Size exclusion chromatograms using UV (top) and RI (bottom) detection: analysis of *i*-Pr--BIAN (black), BOMAG (red), and the mixture of BOMAG with *i*-Pr--BIAN (green).

Table S3. MS identification of the active *i*-Pr-BIAN – alkyl magnesium compounds.

Structure	Alkyl residue -R	t_R / min	m/z observed	m/z calculated
i-Pr-BIAN	-	8.06	501.3268	501.3264
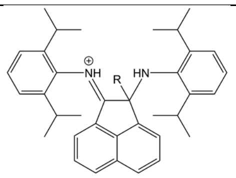	ethyl	7.86	531.3739	531.3734
	butyl	7.79	559.4050	559.4047
	octyl	7.56	615.4688	615.4673

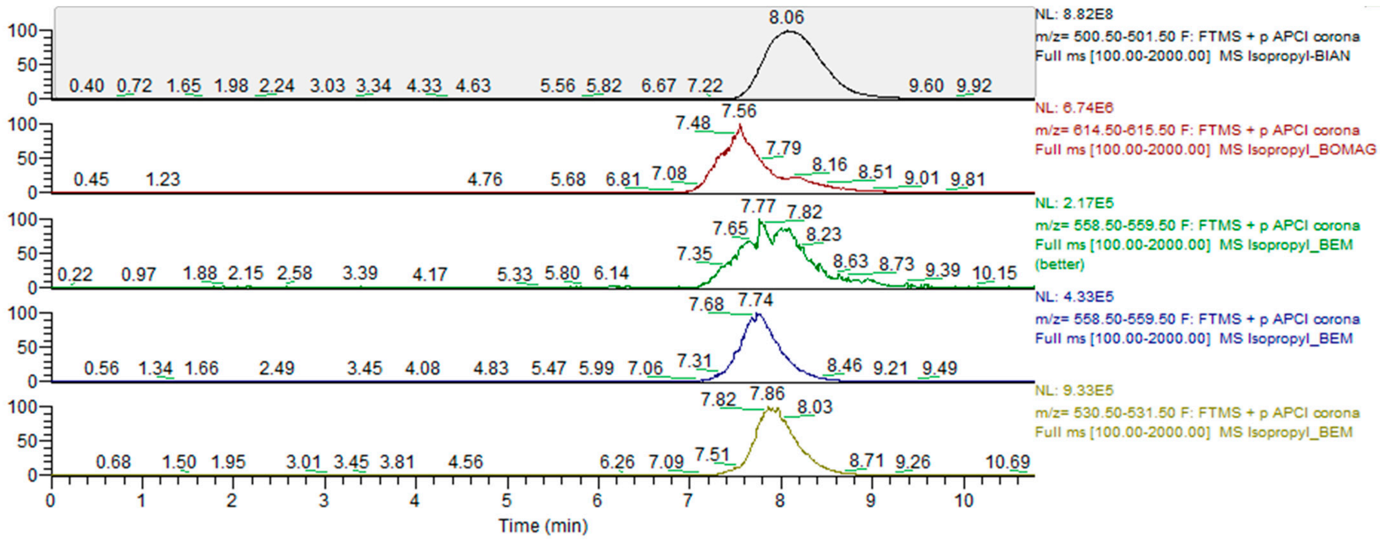

Figure S15. SEC-MS results of *i*-Pr-BIAN. In black pure *i*-Pr-BIAN with m/z= 501.3268 and furthermore the structures formed with *i*-Pr-BIAN with BOMAG and BEM at the following m/z ratios: 615.4688 (red), 559.4050 (green and blue) and 531.3739 (yellow).

3. NMR spectroscopy of the inert reaction mixture of *i*-Prop-BIAN with BOMAG

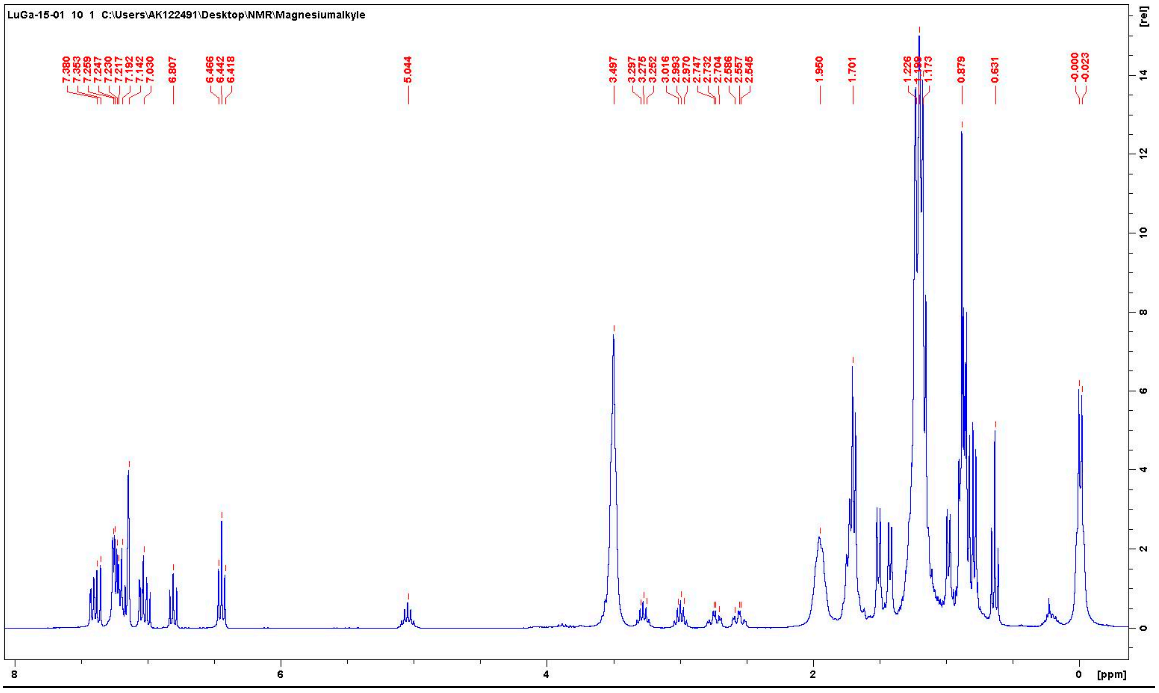

Figure S16. ^{1}H-NMR of *i*-Pr-BIAN modified BOMAG in D_6C_6.

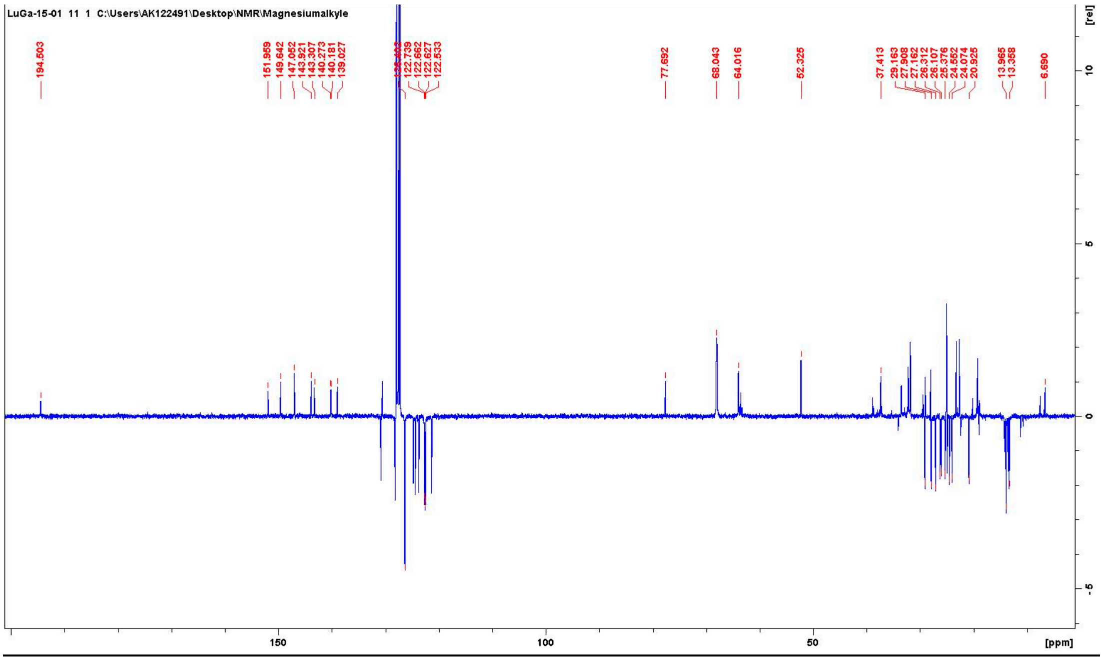

Figure S17. ^{13}C-NMR of *i*-Pr-BIAN modified BOMAG in D_6C_6.

4. HPLC-MS of quenched products

Scheme 1 shows the structures which have been assigned according to high resolution MS data (Table 3). The exact position of the alkyl groups can not always be assigned unequivocally. Also, the exact position of the double bonds cannot be assigned by the experiments carried out in this study.

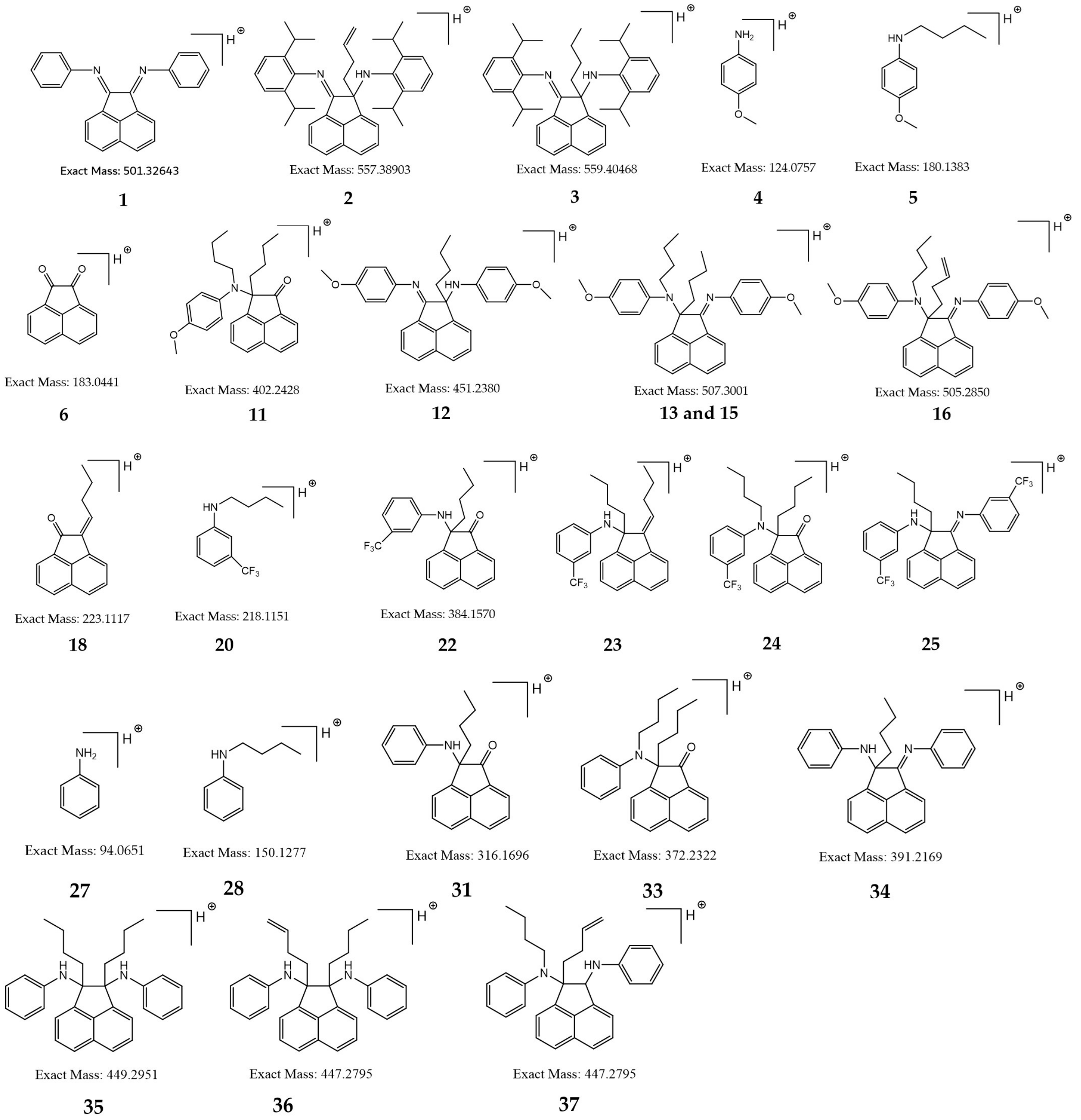

Scheme S1. Tentative structures for the quenched BIAN products with dibutyl magnesium.

5. NMR spectroscopy of quenched BIANs

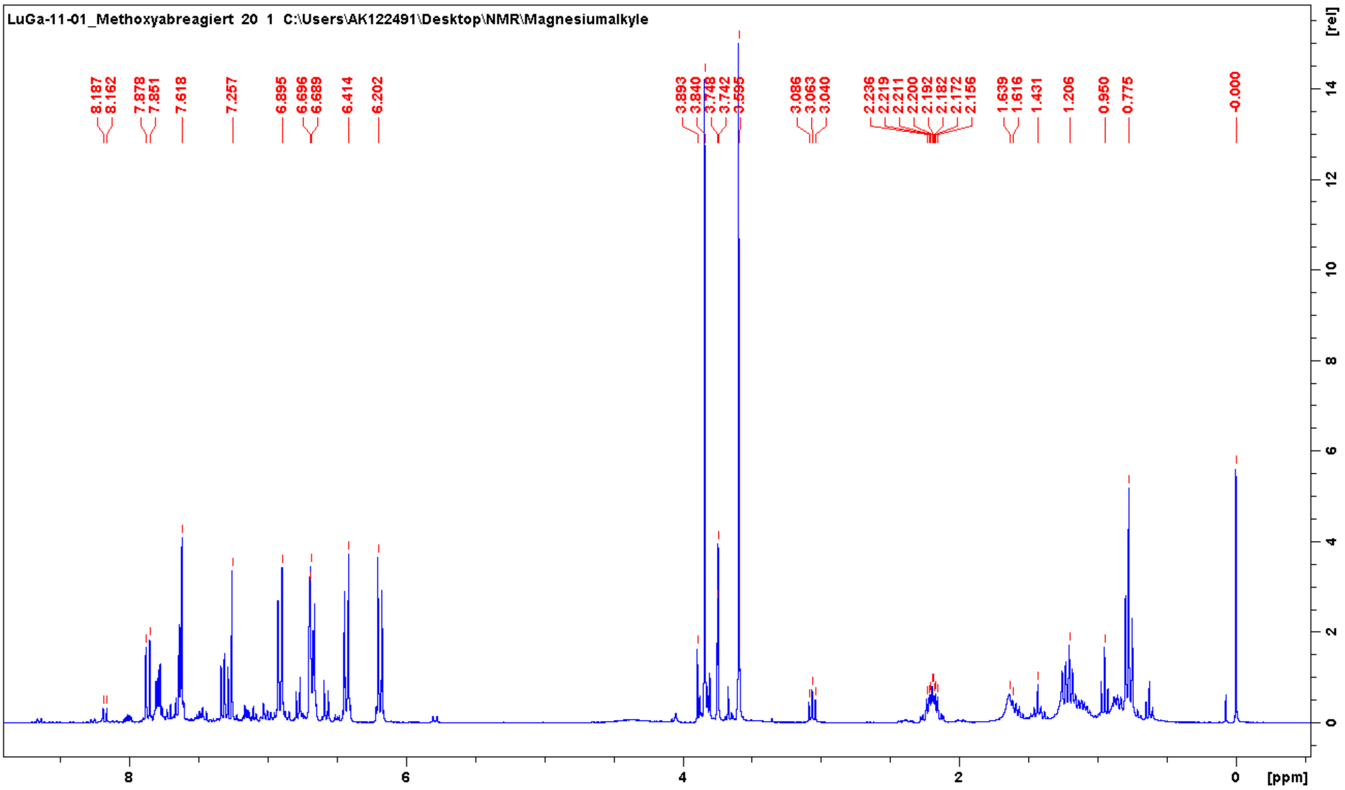

Figure S18. ^{1}H-NMR of quenched MeO-BIAN in $CDCl_3$.

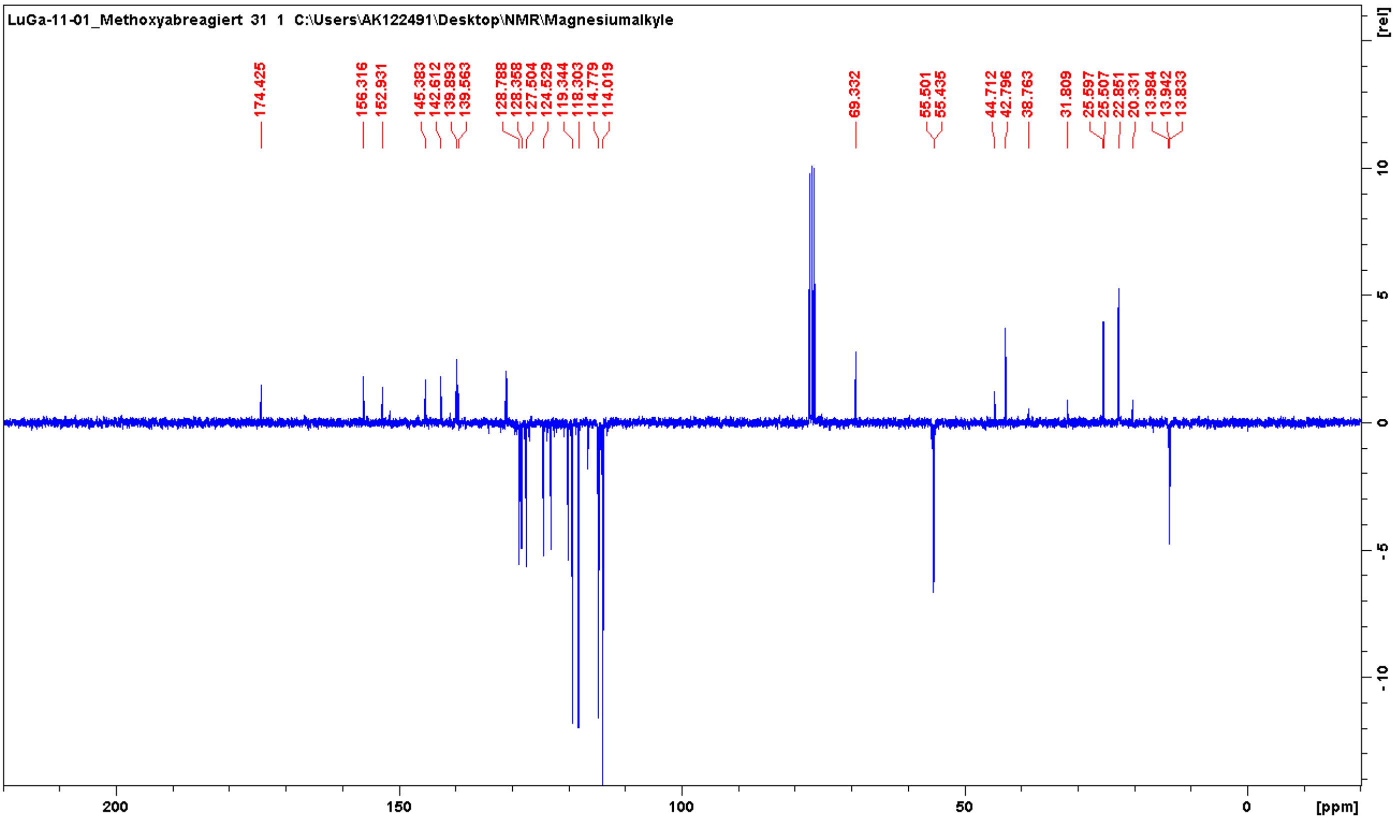

Figure S19. ^{13}C-NMR of quenched MeO-BIAN in $CDCl_3$.

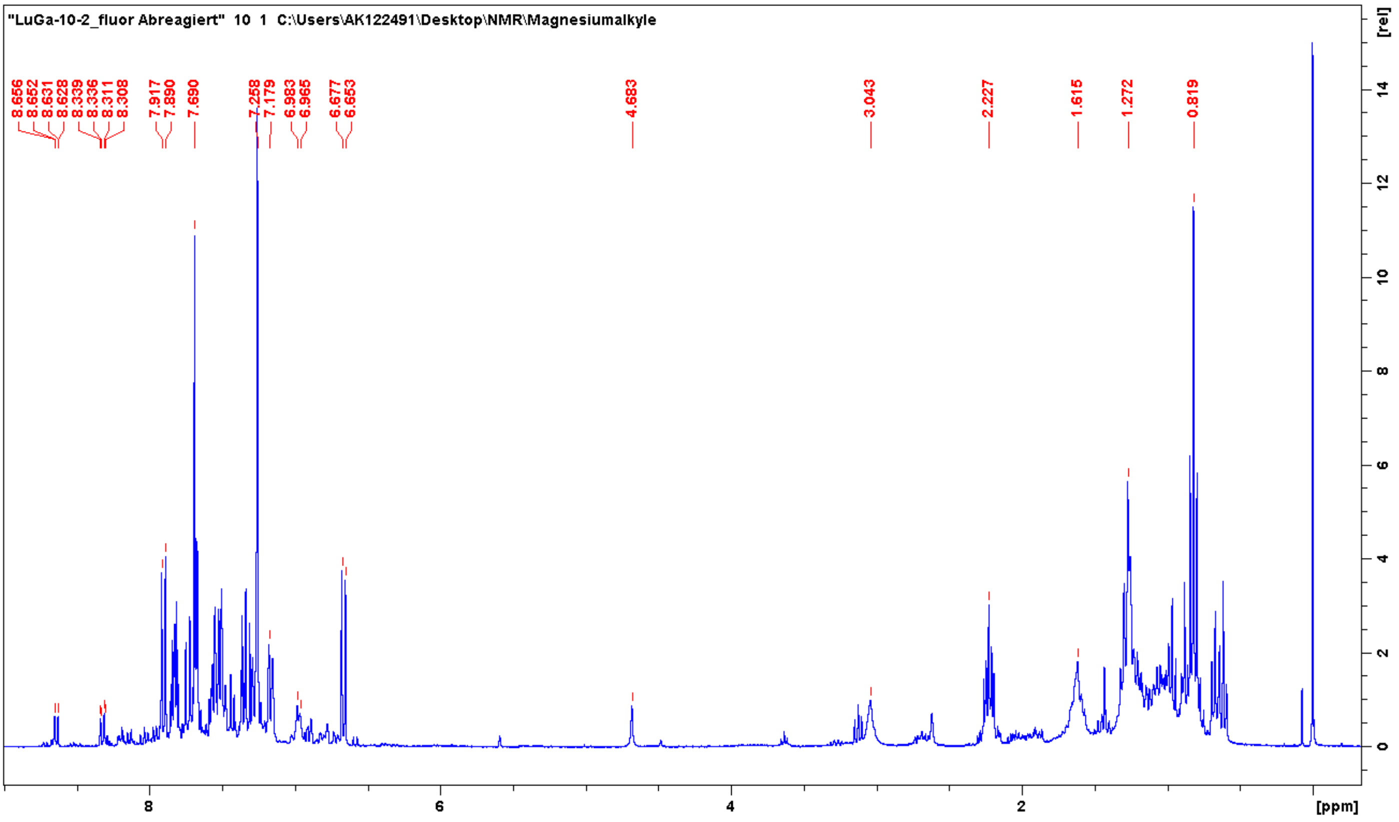

Figure S20. ¹H-NMR of quenched F_3C-BIAN in $CDCl_3$.

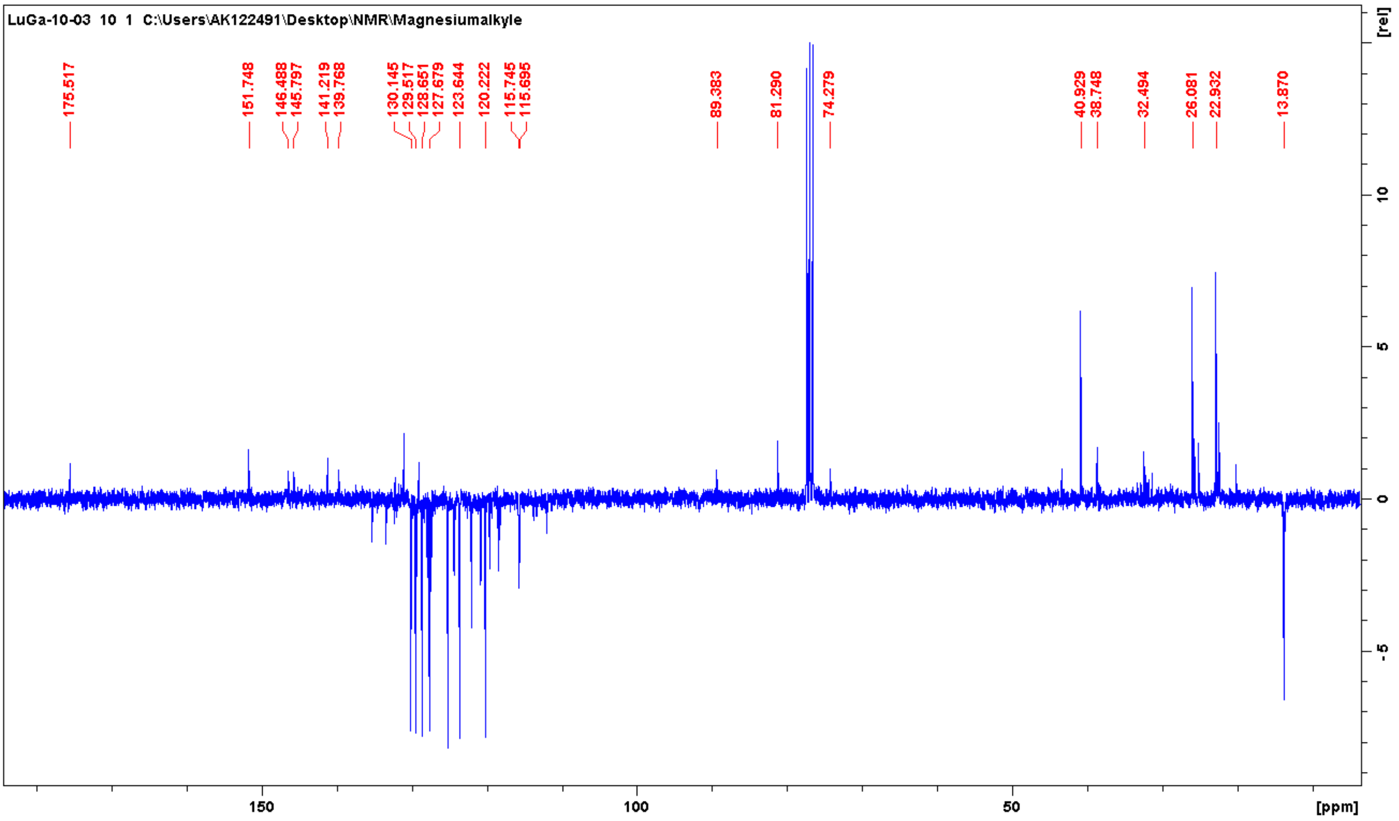

Figure S21. ¹³C-NMR of quenched F_3C-BIAN in $CDCl_3$.
